# Five New Cantharidin Derivatives from the Insect *Mylabris cichorii* L. and Their Potential against Kidney Fibrosis In Vitro

**DOI:** 10.3390/molecules28062822

**Published:** 2023-03-21

**Authors:** Ke-Ming Li, Ji-Jun Li, Li Wan, Yong-Xian Cheng

**Affiliations:** 1 School of Pharmacy, Chengdu University of Traditional Chinese Medicine, Chengdu 611137, China; 2 Institute for Inheritance-Based Innovation of Chinese Medicine, Medical School of Pharmaceutical Sciences, Shenzhen University, Shenzhen 518060, China

**Keywords:** *Mylabris cichorii* linnaeus, insects, cantharidin derivatives, kidney fibrosis

## Abstract

Five new monoterpenoids including three 1-hydroxymethyl-2-methyl cantharimide-type derivatives (**1**, **2**, and **5**) and two 1,2-dimethyl cantharimide-type derivatives (**3** and **4**), together with three known compounds (**6**–**8**) were isolated from the insect *Mylabris cichorii* Linnaeus. The structures of these new compounds, including their absolute configurations, were characterized by detailed analysis of NMR, chemical derivatization, and quantum chemical ECD calculations. All of the compounds were tested for their biological activity against kidney fibrosis. The results revealed that compounds **2**, **4**, and **7** could inhibit kidney fibrosis in vitro at 40 μM by inhibiting the expression of fibronectin and collagen I in TGF-*β*1-induced NRK-52e cells.

## 1. Introduction

**Figure 1:**
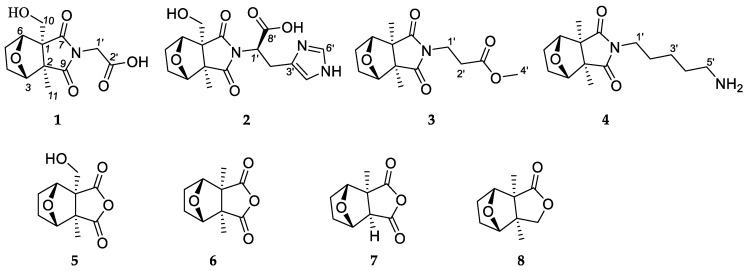
The structures of compounds **1**–**8** from *M. cichorii*.

In recent years, small molecules found in insects have been found to have good biological activities such as antiangiogenic activity [1], renal fibrosis inhibition [2], and COX-2 inhibitory activity [3]. The earliest recording of the blister beetle *Mylabris cichorii* Linnaeus as a medicinal insect is found in Sheng Nong’s *Herbal Classic*. The dried bodies of *M. cichorii* have been used as a traditional Chinese medicine for 2000 years, significantly used for the treatment of tumors [4,5,6,7]. Previous studies have revealed cantharidin and its derivatives as a class of small molecules in the genus *Mylabris* [8,9,10,11]. As a part of our search for characterizing the potent bioactive compounds from insects, research on *M. cichorii* was carried out. As a result, three new 1-hydroxymethyl-2-methyl cantharimide-type cantharidins (**1**, **2**, and **5**), two new 1,2-dimethyl cantharimide-type derivatives (**3** and **4**), and three known compounds (Figure 1) were isolated from *M. cichorii*. Recent studies of monoterpene alkaloids have revealed their powerful anti-fibrotic effects [12,13,14]. Because cantharidin derivatives also resemble such a class of monoterpene alkaloids, we were motivated to conduct a study on kidney fibrosis. Biological evaluation revealed the inhibitory properties of kidney fibrosis thereof. Herein, we report the isolation, structure characterization, and biological evaluation of the isolates.

## 2. Results and Discussion

### 2.1. Structure Elucidation of the Compounds

**Figure 2:**
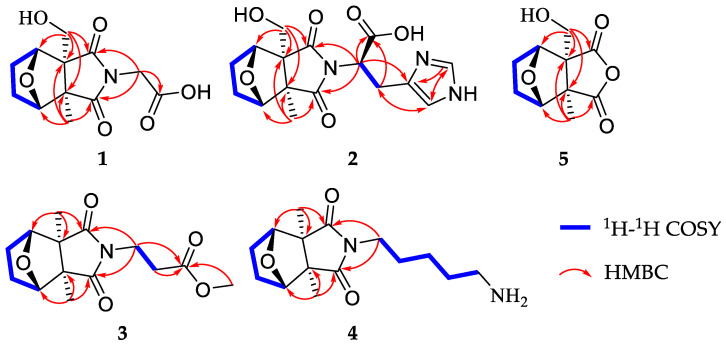
Key ^1^H-^1^H COSY and HMBC correlations of **1**–**5**.

**Figure 3:**
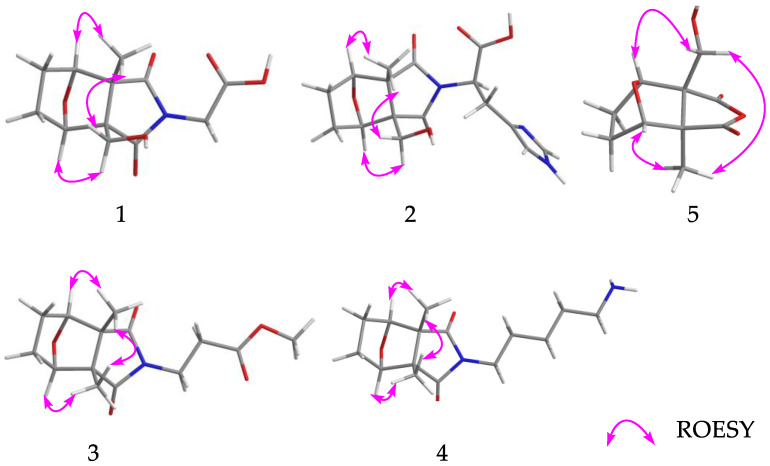
Key ROESY correlations of **1**–**5**.

**Figure 4:**
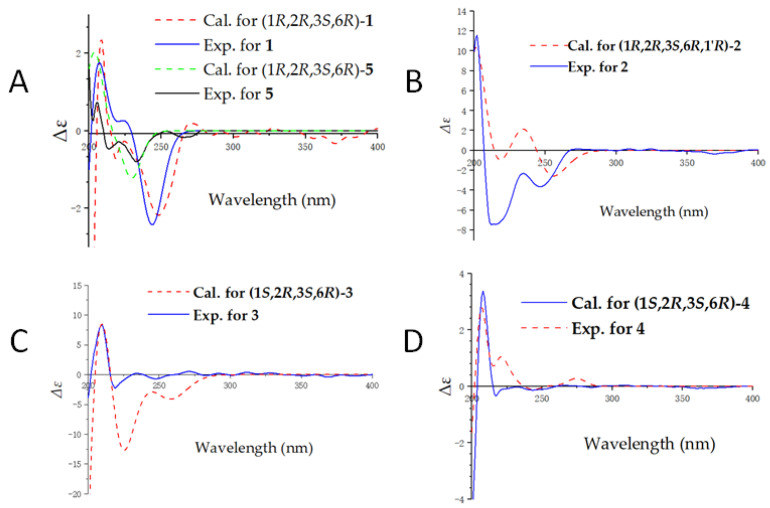
Comparison of the calculated ECD and experimental CD spectra in MeOH. (**A**): The calculated ECD spectrum of (1*R*,2*R*,3*S*,6*R*)-**1** at B3LYP/6-31 (d,p) level, σ = 0.20 eV; shift = −9 nm; The calculated ECD spectrum of (1*R*,2*R*,3*S*,6*R*)-**5** at B3LYP/6-31 (d,p) level, σ = 0.20 eV; shift = −9 nm. (**B**): The calculated ECD spectrum of (1*R*,2*R*,3*S*,6*R*,1′*R*)-**2** at B3LYP/6-31 (d,p) level, σ = 0.30 eV; shift = 0 nm. (**C**): The calculated ECD spectrum of (1*S*,2*R*,3*S*,6*R*)-**3** at B3LYP/6-31 (d,p) level, σ = 0.30 eV; shift = +6 nm. (**D**): The calculated ECD spectrum of (1*S*,2*R*,3*S*,6*R*)-**4** at B3LYP/6-31 (d,p) level, σ = 0.20 eV; shift = +30 nm.

Compound **1***,* isolated as colorless gums, has a molecular formula C_12_H_15_O_6_N (six degrees of unsaturation) based on analysis of the HRESIMS in the positive ion mode, *m/z* 270.0959 [M + H]^+^ (calcd for C_12_H_16_O_6_N, 270.0972) (Figure S7). The UV spectrum of **1** shows an absorption maximum of 203 nm. The ^1^H NMR spectrum of **1** (Table 1, Figure S1) exhibits characteristic signals of cantharidin derivatives, including one methyl group [*δ*_H_ 1.33 (3H, s, H-11)], four methylenes [*δ*_H_ 1.95 (1H, m, Ha-4), 1.67 (1H, m, Hb-4); 1.75 (1H, m, Ha-5), 1.67 (1H, m, Hb-5); 3.97, 3.73 (each 1H, d, *J* = 11.3 Hz); 4.21 (2H, d, *J* = 17.2 Hz)], and two methines [*δ*_H_ 4.52 (1H, br d, *J* = 4.5 Hz), 4.56 (1H, br d, *J* = 4.7 Hz)]. The ^13^C NMR and DEPT spectra (Table 2, Figure S2) exhibit 12 carbon signals including an imide group (*δ*_C_ 182.5, 180.9), a carbonyl group (*δ_C_* 169.7), two oxygenated methines (*δ*_C_ 85.3, 83.2), and two quaternary carbons (*δ*_C_ 61.9, 55.3). The ^1^H and ^13^C NMR data of **1** are similar to cantharimide J [8], except for the signal of one less methylene group at C-2′. The methylene is oxidized as a carbonyl at the C-2′, which could be confirmed by the lower field chemical shift of C-1′ and the ^13^C NMR data of C-2′ at *δ*_C_ 169.9. An HMBC correlation (Figure 2, Figure S4) of H_2_-1′/C-2′ (*δ*_C_ 169.7) further confirmed our conclusion. The relative configuration of **1** was assigned by ROESY correlations (Figure 3, Figure S6), which show H-3 (*δ*_H_ 4.52), H_2_-10 (*δ*_H_ 3.73)/H_3_-11 (*δ*_H_ 1.33), and H_2_-10 (*δ*_H_ 3.97)/H-6 (*δ*_H_ 4.56), showing identical relative configuration at each chiral center with cantharidin. The absolute configuration of **1** was verified by the quantum chemical electronic circular dichroism (ECD) calculations. It was found that the experimental ECD spectrum of **1** is similar to the calculated one of (1*R*,2*R*,3*S*,6*R*)-**1** (Figure 4, Figure S8). Thus, the structure of **1** was finally deduced and named cichormide A.

Compound **2** was determined to have a molecular formula of C_16_H_19_O_6_N_3_ from its HRESIMS (*m/z* 350.1347 [M + H]^+^, C_16_H_20_O_6_N_3_, 350.1360) (Figure S15), indicating the presence of nine degrees of unsaturation. The ^1^H NMR data (Table 1, Figure S9) of **2** show one methyl [*δ*_H_ 1.24 (3H, s, H-11)], four methylenes [*δ*_H_ 3.94 (1H, d, *J* = 11.2 Hz, Ha-10), 3.69 (1H, d, *J* = 11.2 Hz, Hb-10), 1.62-1.91 (4H, m, H_2_-4, H_2_-5), *δ*_H_ 3.49 (2H, br d, *J* = 8.0 Hz, H-2′)], and five methines [*δ*_H_ 8.71 (1H, s, H-6′), 7.26 (1H, s, H-4′), 4.97 (1H, br t, *J* = 7.4 Hz, H-1′), *δ*_H_ 4.46 (1H, br d, *J* = 4.6 Hz, H-3), 4.36 (1H, br d, *J* = 4.8 Hz, H-6)]. The ^13^C NMR and DEPT spectra (Table 2, Figure S10) show 16 carbon signals, which were assigned as one methyl, four methylenes, five methines, and six non-protonated carbons (including 2 carbonyls and 1 carboxyl). These featured NMR signals are similar to those of canthaminomide F [11], suggesting that **2** is a 1-hydroxymethyl-2-methyl cantharimide-type derivative. The key difference is that the substitution of a methyl at C-1 is replaced by a hydroxymethyl, which is supported by the HMBC correlations of H_2_-10/C-2, C-6, and C-7 (Figure 2, Figure S12). Thus, the planar structure of **2** was assigned. The ROESY correlations between H-3 (*δ*_H_ 4.46), H_2_-10 (*δ*_H_ 3.69)/H_3_-11 (*δ*_H_ 1.24), and between H_2_-10 (*δ*_H_ 3.94)/H-6 (*δ*_H_ 4.36) suggested that **2** possesses the same relative configuration as compound **1** in the counterpart (Figure 3, Figure S14). The resolution of the configuration at C-1′ has been reported in previous literature [11], and the same method was used to determine the configuration of compound **2**. The D or L-histidine was, respectively, submitted to a reaction containing compound **5** under 95% EtOH of solvent at 78 °C for 48 h, which led to the generation of **2a** (1*R*,2*R*,3*S*,6*R*,1′*R*)/**2b** (1*R*,2*R*,3*S*,6*R*,1′*S*) (Supplementary Materials, Scheme S1). A careful comparison of the NMR data and the retention time in the HPLC chromatogram of **2a** and **2b** with those of compound **2** revealed that **2a** is actually the same as **2** (Supplementary Materials, Figures S41–S45). Thus, the absolute configuration of **2** at C-1′ was assigned. With this information in hand, the absolute configurations at the rest chiral carbons of **2** were solved by comparison of the theoretical and experimental ECD spectra (Figure 4, Figure S16). The results showed that the calculated ECD spectrum of (1*R*,2*R*,3*S*,6*R*,1′*R*)-**2** is in good accordance with that of **2**. Thus, the structure of **2** was determined to be [(1*R*,2*R*,3*S*,6*R*,1′*R*)- 1-hydroxymethyl-2-methyl-3,6-epoxycyclohexane-1,2-dicarboximide]-(1′*R*)-histidine and named cichormide B.

Compound **3** has a molecular formula of C_14_H_19_O_5_N by the HRESIMS at *m/z* 282.1338 [M + H]^+^ in a combination of the NMR data, indicating six degrees of unsaturation (Figure S23). The UV spectrum of **3** shows an absorption maximum of 204 nm. The ^1^H NMR data of **3** (Table 1, Figure S17) show signals for two methyls [*δ*_H_ 1.15 × 2 (6H, s)], four methylenes [*δ*_H_ 3.74 (2H, t, *J* = 7.0 Hz), 2.58 (2H, t, *J* = 7.0 Hz), 1.89 (2H, overlapped), 1.64 (2H, overlapped)], two methines [*δ*_H_ 4.47 × 2 (1H, overlapped)], and one methoxy group [*δ*_H_ 3.64 (3H, s)]. The ^13^C NMR and DEPT spectra (Table 2, Figure S18) exhibit 14 signals including an imide group [*δ*_C_ 183.1 × 2], two oxygenated methines [*δ*_C_ 85.1 × 2], a carbonyl group (*δ*_C_ 172.9), and two quaternary carbons (*δ*_C_ 55.3 × 2). The ^13^C NMR spectrum of **3** is similar to that of cantharimide E [8], except for one additional methoxy group at *δ*_C_ 52.4. The amine group in cantharimide E at C-3′ is absent in **3** and replaced by a methoxy group, which could be further confirmed by the HMBC (Figure 2, Figure S20) correlation between H_3_-4′ and the carbonyl group *δ*_C_ 172.9 (C-3′). In the same manner, the relative configuration of **3** was determined by ROESY correlations (Figure 3, Figure S22) of H_3_-10 or H_3_-11/H-3 (*δ*_H_ 4.47), H-6 (*δ*_H_ 4.48), suggesting that **3** possesses the same relative configuration as that of cantharidin. Likewise, the absolute configuration of **3** was determined as (1*S*,2*R,*3*S*,4*R*)-**3** by comparing the calculated ECD spectrum with the experimental one (Figure 4, Figure S24). Taken together, the structure of **3** was finally identified and named cichormide C.

Compound **4** was obtained as yellow gums. The molecular formula of **4** was deduced as C_15_H_24_O_3_N_2_ by its HRESIMS at *m/z* 281.1851 [M + H]^+^ (calcd for C_15_H_25_O_3_N_2_, 281.1860) (Figure S31), indicating five degrees of unsaturation. The UV spectrum of **4** shows an absorption maximum of 204 nm. In the ^1^H NMR spectrum of **4** (Table 1, Figure S25), two methyls [*δ*_H_ 1.15 × 2 (6H, s)], seven methylenes [*δ*_H_ 3.51 (2H, t, *J* = 7.0 Hz), 2.89 (2H, t, *J* = 7.7 Hz), 1.91 (2H, overlapped), 1.66 (4H, overlapped), 1.61 (2H, m), 1.36 (2H, m)], and two methines [*δ*_H_ 4.47 × 2 (1H, overlapped)] were observed. The ^13^C NMR and DEPT spectra (Table 2, Figure S26) exhibit 15 signals including an imide group [*δ*_C_ 183.6 × 2], two oxygenated methines [*δ*_C_ 85.2 × 2], and two quaternary carbons (*δ*_C_ 55.2 × 2). The ^13^C NMR data of **4** are similar to those of (2*S*)-6-amino-2-[(3aR*,4S*,7R*,7aS*)-3a,7a-dimethyl-1,3-dioxo-4,7-epoxyoctahydroisoindol-2-yl]-hexanoic acid [9], except for one disappeared carbonyl group at C-1′, which could be confirmed by the ^1^H-^1^H COSY correlations of H_2_-1′/H_2_-2′/H_2_-3′/H_2_-4′/H_2_-5′ and HMBC correlation of H_2_-1′/C-7, C-9 (Figure 2, Figures S28 and 29). The ROESY correlations (Figure 3) of H_3_-10 or H_3_-11 (*δ*_H_ 1.15)/H-3 (*δ*_H_ 4.47), and H-6 (*δ*_H_ 4.48), suggested that **4** possesses the same relative configuration as **3** (Figure S30). The absolute configuration of **4** was assigned as (1*S*,2*R,*3*S,*4*R*)-**4** by ECD calculations (Figure 4, Figure S32). Thus, the structure of compound **4** was finally identified and named cichormide D.

Compound **5** was isolated as white powders. Its molecular formula was deduced as C_10_H_12_O_5_ by analysis of its positive HRESIMS, ^13^C NMR, and DEPT spectra, indicating five degrees of unsaturation (Figure S39). The ^1^H NMR spectrum of **5** (Table 1, Figure S33) gives a methyl [*δ*_H_ 1.41 (3H, s, H-11)], three methylenes [*δ*_H_ 3.98 (1H, d, *J* = 11.1 Hz, Ha-10), 3.72 (1H, d, *J* = 11.1 Hz, Hb-10), 1.68-1.96 (4H, m, H_2_-4, H_2_-5)], and two methines [*δ*_H_ 4.67 (1H, br d, H-3), *δ*_H_ 4.66 (1H, br d, H-6)]. The ^13^C NMR and DEPT spectra (Table 2, Figure S34) display 10 signals including an anhydride group [*δ*_C_ 177.9, 176.0], two oxygenated methines [*δ*_C_ 86.7, 84.2], and two quaternary carbons [*δ*_C_ 64.3, 55.9]. Compound **5** bears the same carbon skeleton as (1*R*,2*R*,3*S*,6*R*)-1-hydroxymethyl-2-methyl-3,6-epoxycyclohexane-1,2-dicarboximide [15] by inspection of their NMR spectra. The only difference between them is that position 8 is an oxygen atom instead of a nitrogen atom, which is supported by the chemical shifts of C-7 (*δ*_C_ 176.0) and C-9 (*δ*_C_ 177.9) shifting to the upfield 7 ppm. In addition, the ROESY correlations (Figure 3, Figure S38) between H_2_-10 (*δ*_H_ 3.72) and H-3 (*δ*_H_ 1.67)/H_3_-11 (*δ*_H_ 1.41), H_2_-10 (*δ*_H_ 3.98)/H-6 (*δ*_H_ 4.66) suggested that **5** possesses the same relative configurations as cantharidin. In the end, the absolute configuration of **5** was determined as (1*S*,2*R,*3*S*,4*R*)-**5** by comparing the calculated ECD spectrum with those of compounds **1** and **2** (Figure 4, Figure S40). Thus, the structure of **5** was finally identified and named 10-hydroxy-cantharidin.

As mentioned above, cantharidin derivatives have been characterized by the genus *Mylabris*. It is clear that the side chain attaching to the nitrogen atom could be different amino acid residues. In this study, the side chains belonging to glycine, histidine, alanine, and lysine were observed adding the diversity for the side chain of suan a class of compounds.

The known compounds were identified as cantharidin (**6**) [16], palasonin (**7**) [17], and 2.6-dimethy1-4,10-dioxa-3-oxo-tricyclo [5.2.1.0^2,6^]decane (**8**) [18], respectively, by comparing their spectroscopic data with those reported in the literature.

### 2.2. Biological Evaluation

**Figure 5:**
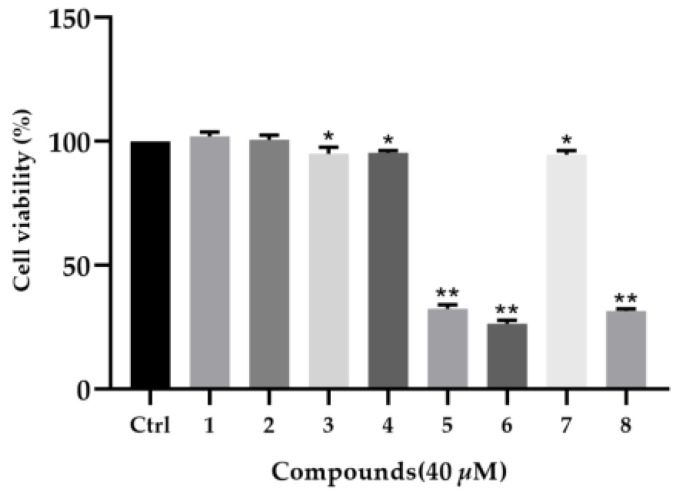
NRK-52e cell proliferation in response to compounds at 40 μM by CCK-8 assay. * *p* < 0.05, ** *p* < 0.01 compared with Control alone.

**Figure 6:**
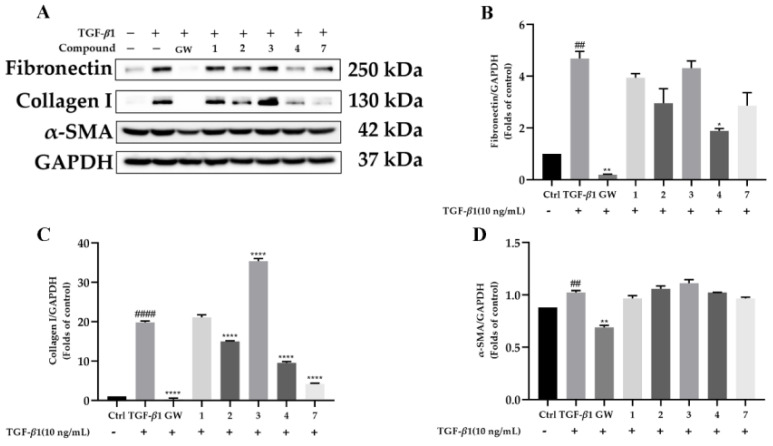
Compounds attenuate renal fibrosis in TGF-*β*1-induced NRK-52e cells. Cells were incubated in different concentrations of the compound and then exposed to 10 ng/mL TGF-*β*1 for 48 h. (**A**–**D**): The protein level of Fibronectin, Collagen I, and *α*-SMA in NRK-52e were determined by Western blotting, and GAPDH was used as a control. Data represent mean ± SEM values of three experiments. * *p* < 0.05, ** *p* < 0.01 and **** *p* < 0.0001 compared with TGF-*β*1 alone. ## *p* < 0.01 and #### *p* < 0.0001 compared with Control alone. GW788388 (GW) was used as a positive control.

**Figure 7:**
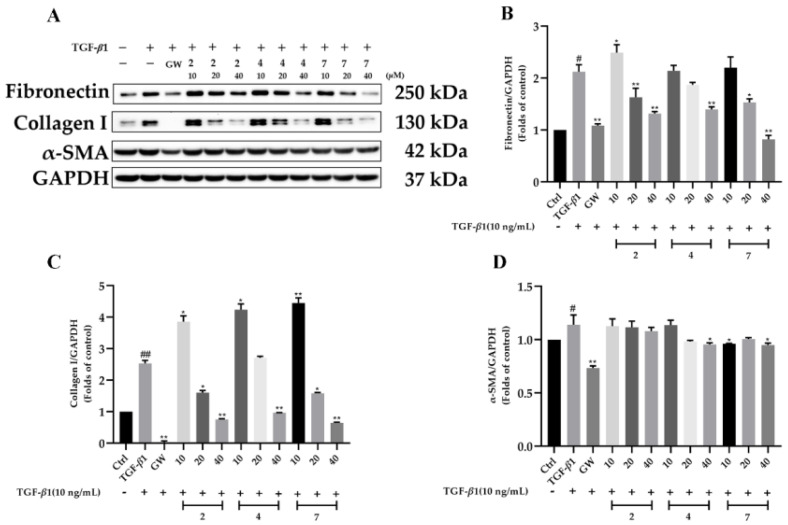
Compounds attenuate renal fibrosis in TGF-*β*1-induced NRK-52e cells. NRK-52e cells were incubated with TGF-*β*1 (10 ng/mL) for 48 h in the absence or presence of different concentrations (10 μM, 20 μM, and 40 μM) of compounds **2**, **4**, and **7**. (**A**–**D**): The protein level of Fibronectin, Collagen I, and *α*-SMA in NRK-52e were determined by Western blotting, and GAPDH was used as a control. Data represent mean ± SEM values of three experiments. * *p* < 0.05, ** *p* < 0.01 compared with TGF-*β*1 alone. # *p* < 0.05 and ## *p* < 0.01 compared with Control alone. GW788388 (GW) was used as a positive control.

The renal protection of all the isolates was carried out in TGF-*β*1-induced rat renal proximal tubular cells. To exclude the possibility that the biological effects of the compounds are caused by cytotoxicity, a CCK-8 assay was first carried out (Figure 5). The results showed that the other compounds had slight toxicity toward rat renal proximal tubular cells (NRK-52e) except for **5**, **6**, and **8**. Hence, the renal protection property of compounds **1–4** and **7** were evaluated (Figure 6). As presented in Figure 7, compounds **2**, **4**, and **7** were found to reduce the expression of fibronectin and collagen I in a dose-dependent manner in TGF-*β*1-induced NRK-52e cells. Since fibronectin and collagen I are components of the extracellular matrix and overexpression of the extracellular matrix is considered to be the hallmark of renal fibrosis, our current finding disclosed that cantharidin derivatives might be potent agents in renal protection. To our knowledge, this is the first time that cantharidin derivatives have been found to possess biological activity in renal fibrosis.

## 3. Experimental Section

### 3.1. General Procedures

Optical rotations were recorded on an Anton Paar MCP-100 digital polarimeter. UV and CD spectra were measured on a Chirascan instrument (Agilent Technologies, Santa Clara, CA, USA). NMR spectra were collected by a Bruker Avance III 600 MHz or a 500 MHz spectrometer, and the internal standard was TMS. HRESIMS were recorded on a Shimazu LC-20AD AB Sciex triple X500R MS spectrometer (Shimadzu Corporation, Tokyo, Japan). Macroporous adsorbents (Rohmhass AMBERLITE^TM^ XAD 16N, America), MCI gel CHP 20P (75–150 μm, Mitsubishi Chemical Industries, Tokyo, Japan), chromatography. YMC gel ODS-A-HG (40–60 μm; YMC Co., Tokyo, Japan), Sephadex LH-20 (Amersham Pharmacia, Uppsala, Sweden), and Silica gel (200–300 mesh, Qingdao Marine Chemical Inc., Qingdao, China) were used for column chromatography. Preparative HPLC was carried out using a Chuangxin-Tongheng chromatograph equipped with a Thermo Hypersil GOLD-C_18_ column (250 mm × 21.2 mm, i.d., 5 μm). Semi-preparative HPLC was taken on a SEP-LC52 chromatograph with a YMC-Pack ODS-A column (250 mm × 10 mm, i.d., 5 μm). Racemic compounds were purified by chiral HPLC on a Daicel Chiralpak column (IC, 250 mm × 4.6 mm, i.d., 5 μm) or a Phenomenex column (OOG-4762-E0 LUX^®^ i-Amylose-1, 250 mm × 4.6 mm, i.d., 5 μm) at a flow rate of 1.0 mL/min.

### 3.2. Insect Material

*M. cichorii* were collected from Henan province, China, in July 2020, and identified by Prof. Dang-Rong Yang from the Kunming Institute of Zoology, Chinese Academy of Sciences, Kunming, China. A voucher specimen (CHYX-0643) was deposited at the School of Pharmaceutical Sciences, Shenzhen University Health Science Center, China.

### 3.3. Extraction and Isolation

The air-dried powdered *M. cichorii* (9 kg) were extracted with 50% aqueous EtOH (4 *×* 45 L, 24 h each) at room temperature. The combined extracts were concentrated to obtain a crude extract (1.5 kg), which was then divided into six parts (Fr.A–Fr.F) by using a macroporous adsorbents Rohmhass AMBERLITE^TM^ XAD 16N column eluted with gradient aqueous EtOH (0:100–100:0). Fr.B (20.0 g) was isolated by an MCI gel CHP 20P column eluted with gradient aqueous MeOH (3–100%) to afford five fractions (Fr.B1–Fr.B5). Fr.B1 (3.2 g) was subjected to Sephadex LH-20 (MeOH) to afford eight fractions (Fr.B1.1–Fr. B1.8). Fr.B1.3 (310.0 mg) was separated by preparative HPLC (MeCN/H_2_O with 0.05% TFA, 1–50%, flow rate: 8 mL/min) to yield thirteen portions (Fr.B1.3.1–Fr.B1.3.13). Fr.B1.3.4 (71.0 mg) was purified by semi-preparative HPLC (MeCN/H_2_O with 0.05% TFA, 7%, flow rate: 3 mL/min) to obtain compound **2** (*t*_R_ = 10.8 min, 53.1 mg). Fr.B2 (2.8 g) was subjected to Sephadex LH-20 (MeOH) to afford six fractions (Fr.B2.1–Fr.B2.6). Fr.B2.2 (295.0 mg) was separated by preparative HPLC (MeCN/H_2_O with 0.05% TFA, 1–50%, flow rate: 8 mL/min) to yield six portions (Fr.B2.2.1–Fr.B2.2.6). Fr.B2.2.6 (63.1 mg) was purified by semi-preparative HPLC (MeCN/H_2_O with 0.05% TFA, 12%, flow rate: 3 mL/min) to obtain compound **1** (*t*_R_ = 15.5 min, 16.1 mg). Fr.B4 (1.0 g) was subjected to Sephadex LH-20 (aqueous MeOH, 70%) to afford ten fractions (Fr.B4.1–Fr.B4.10). Fr.B4.8 (61.4 mg) was purified by semi-preparative HPLC (MeCN/H_2_O with 0.05% TFA, 11%, flow rate: 3 mL/min) to obtain compound **7** (*t*_R_ = 23.0 min, 4.1 mg). Fr.C (180.0 g) was separated into seven fractions (Fr.C1–Fr.C7) by MCI gel CHP 20P column chromatography (aqueous MeOH, 10–100%). Fr.C2 (64.0 g) was subjected to an ODS column on an MPLC system eluted with gradient aqueous MeOH (5–100%, flow rate: 20 mL/min) to afford five fractions (Fr.C2.1–Fr.C2.5). Fr.C2.2 (13.0 g) was subjected to Sephadex LH-20 (MeOH) to afford compound **5** (2.3 g) and eight fractions (Fr.C2.2.1–Fr.C2.2.8). Fr.C2.2.1 (210.1 mg) was separated by preparative HPLC (MeCN/H_2_O with 0.05% TFA, 5–100%, flow rate: 8 mL/min) to yield four portions (Fr.C2.2.1.1–Fr.C2.2.1.4). Fr.C2.2.1.2 (47.0 mg) was purified by semi-preparative HPLC (MeCN/H_2_O with 0.05% TFA, 17%, flow rate: 3 mL/min) to obtain compound **4** (*t*_R_ = 20.3 min, 4.7 mg). Fr.C2.2.4 (1.3 g) was separated into six fractions (Fr.C2.2.4.1–Fr.C2.2.4.6) by Sephadex LH-20 (aqueous MeOH, 70%). Fr.C2.2.4.1 (180.3 mg) was separated by preparative HPLC (MeOH/H_2_O with 0.05% TFA, 10–100%, flow rate: 8 mL/min) to yield four portions (Fr.C2.2.4.1.1–Fr.C2.2.4.1.4). Fr.C2.2.4.1.3 (72.5 mg) was purified by semi-preparative HPLC (MeOH/H_2_O with 0.05% TFA, 26%, flow rate: 3 mL/min) to obtain compound **3** (*t*_R_ = 30.5 min, 2.3 mg). Fr.C2.3 (6.2 g) was separated into six fractions (Fr.C2.3.1–Fr.C2.3.6) by Sephadex LH-20 (MeOH). Fr.C2.3.4 (479.4 mg) was separated by preparative HPLC (MeOH/H_2_O with 0.05% TFA, 10–100%, flow rate: 8 mL/min) to yield nine portions (Fr.C2.3.4.1–Fr.C2.3.4.9). Fr.C2.3.4.6 (77.2 mg) was purified by semi-preparative HPLC (MeCN/H_2_O with 0.05% TFA, 20%, flow rate: 3 mL/min) to obtain compound **8** (*t*_R_ = 22.7 min, 3.1 mg). Fr.F (90.0 g) was separated into ten fractions (Fr. F1–Fr.F10) by MCI gel CHP 20P column chromatography (aqueous MeOH, 60–100%). Fr.F4 (15.0 g) was subjected to Sephadex LH-20 (MeOH) to afford six fractions (Fr.F4.1–Fr.F4.6). Fr.F4.4 (2.3 g) was fractionated into eight parts (Fr.F4.4.1–Fr.F4.4.8) by a silica gel column eluted by increasing ethyl acetate in petroleum ether (10:1–3:1). Fr.F4.4.4 (1.8 g) was gel filtrated over Sephadex LH-20 (MeOH) to get compound **6** (1.5 g).

### 3.4. Compound Characterization Data

*Cichormide A* (**1**): Colorless gum, [α${]}_{D}^{25}$ –0.6 (*c* 0.34, MeOH); CD (MeOH) Δ*ε*_48_ –0.36, Δ*ε*_205_ +0.63, Δ*ε*_200_ –2.26; UV (MeOH) *λ*_max_ (log*ε*) 203 (2.67) nm; HRESIMS *m/z* 270.0959 [M + H]^+^, (calcd for C_12_H_16_O_6_N, 270.0972). ^1^H and ^13^C NMR data, see Table 1 and Table 2.

*Cichormide B* (**2**): Yellow gum, [α${]}_{D}^{25}$ –40.3 (*c* 0.39, MeOH); CD (MeOH) Δ*ε*_268_ +0.01, Δ*ε*_246_ –0.66, Δ*ε*_235_ –0.42, Δ*ε*_212_ –1.36, Δ*ε*_202_ +2.10; UV (MeOH) *λ*_max_ (log*ε*) 202 (2.49) nm; HRESIMS *m/z* 350.1347 [M + H]^+^, (calcd for C_16_H_20_O_6_N_3_, 350.1329). ^1^H and ^13^C NMR data, see Table 1 and Table 2.

*Cichormide C* (**3**): Colorless gum, [α${]}_{D}^{25}$ –0.6 (*c* 0.35, MeOH); CD (MeOH) Δ*ε*_219_ –0.19, Δ*ε*_209_ +0.17, Δ*ε*_200_ –0.33; UV (MeOH) *λ*_max_ (log*ε*) 204 (2.71) nm; HRESIMS *m/z* 282.1338 [M + H]^+^, (calcd for C_14_H_20_O_5_N, 282.1336). ^1^H and ^13^C NMR data, see Table 1 and Table 2.

*Cichormide D* (**4**): Yellow gum, [α${]}_{D}^{25}$ +0.3 (*c* 0.37, MeOH); CD (MeOH) Δ*ε*_217_ –0.18, Δ*ε*_208_ +1.79, Δ*ε*_200_ –2.57; UV (MeOH) *λ*_max_ (log*ε*) 204 (2.68) nm; HRESIMS *m/z* 281.1851 [M + H]^+^, (calcd for C_15_H_25_O_3_N_2_, 281.1860). ^1^H and ^13^C NMR data, see Table 1 and Table 2.

*10-hydroxy-cantharidin* (**5**): white powder, [α${]}_{D}^{25}$ 0 (*c* 0.30, MeOH); CD (MeOH) Δ*ε*_200_ +0.15; UV (MeOH) *λ*_max_ (log*ε*) 200 (0.43) nm; HRESIMS *m/z* 212.0759 [M + H]^+^, (calcd for C_10_H_13_O_5_, 211.0757). ^1^H and ^13^C NMR data, see Table 1 and Table 2.

### 3.5. Computational Methods

Molecular Merck force field (MMFF) and DFT/TDDFT were calculated with the Spartan’14 software package and Gaussian 09 program package. Electronic circular dichroism (ECD) calculations were conducted at the B3LYP/6-31g(d,p) level and the CD spectra were produced by the program SpecDis 1.62 (Figures S46–S50 and Tables S1–S5) [19].

### 3.6. Kidney Fibrosis Activity

#### 3.6.1. Cell Culture

NRK-52e, rat renal proximal tubular cells (Cell Bank of China Science Academy, Shanghai, China) were cultured in high-glucose DMEM (C11995500BT, Gibco, Waltham, MA, USA) supplemented with 10% fetal bovine serum (FBS) (2094468CP, Gibco, Waltham, MA, USA), 100 U/mL penicillin, and 100 μg/mL streptomycin at 37 °C in a humidified environment containing 5% CO_2_.

#### 3.6.2. Cell Viability Assay

NRK-52e (1× 10^4^ cells/mL) cells were seeded into a 96-well plate with completed DMEM. After overnight culture, cells were treated with various concentrations of compounds or DMSO for 48 h. Then, Cell Count Kit-8 (CCK-8, Beyotime, Shanghai, China) was added into each well for 1 h at 37 °C. The absorbance of each well was recorded at 450 nm using a microplate reader (BioTek, Winooski, VT, USA).

#### 3.6.3. Western Blot

NRK-52e cells were treated with TGF-β1 (10 ng/mL) for 48 h in the absence or presence of 40 μM compounds. Cell lysates were prepared with RIPA buffer (Beyotime, Shanghai, China) containing 1 × protease inhibitor cocktail (Roche, Mannheim, Germany), 1 × phosphatase inhibitor cocktails, 0.1 mM PMSF, and quantified protein samples using the BCA assay (Thermo Scientific, Waltham, MA, USA). Equal amounts of protein extracts were separated by 8% SDS-PAGE and transferred to PVDF membranes (Millipore, Darmstadt, Germany). The membranes were blocked with 5% BSA, then with the indicated antibodies overnight at 4 °C, and were then followed by incubation with horseradish peroxidase (HRP)-conjugated secondary antibody at room temperature. The bands were visualized and measured via the ECL kit (Pierce, Hercules, CA, USA) and analysis system (Bio-Rad, Hercules, CA, USA). The primary antibodies are as follows: Anti-fibronectin antibody [IST-9] (#ab6328; Abcam, Cambridge, UK), Col1A1 antibody (#84336; Cell Signaling Technology, Boston, MA, USA), α-SMA (D4K9N) XP*^®^* Rabbit mAb (#19245, Cell Signaling Technology, Boston, MA, USA), and GAPDH (D16H11) XP*^®^* Rabbit mAb (#5174, Cell Signaling Technology, Boston, MA, USA).

## 4. Conclusions

In conclusion, our study resulted in the characterization of cantharidin derivatives from the title material, adding new facets for cantharidin structure diversity. Furthermore, biological comparison found that cantharidin derivatives with an oxygen atom instead of a nitrogen atom at position 8 and an alkyl group at C-1 and C-2 are toxic. To date, a limited number of cantharidin derivatives (<50) have been investigated from the *Mylabris* species and their potential for kidney fibrosis has not been described. Finally, the anti-fibrotic activity of cantharimide-type derivatives might provide new insight into the biological profiling of chemicals from *M. cichorii*, an excellent alternative resource for new pharmaceuticals.

## Figures and Tables

**Table 1:** The ^1^H NMR (600 MHz) data of **1–5** in CD_3_OD (*δ* in ppm, *J* in Hz).

Position	1	2	3	4	5
3	4.52 (br d, 4.5)	4.46 (br d, 4.6)	4.47 (br d, 2.6)	4.47 (br d, 2.2)	4.67 (br d, 2.7)
4	Ha: 1.95 (m)	Ha: 1.91 (m)	Ha: 1.89 (overlap) ^a^	Ha: 1.91 (overlap) ^a^	Ha: 1.97 (m)
	Hb: 1.67 (overlap) ^a^	Hb: 1.61 (overlap) ^a^	Hb: 1.64 (overlap) ^a^	Hb: 1.65 (overlap) ^a^	Hb: 1.68 (overlap) ^a^
5	Ha: 1.75 (m)	Ha: 1.67 (m)	Ha: 1.89 (overlap) ^a^	Ha: 1.91 (overlap) ^a^	Ha: 1.68 (overlap) ^a^
	Hb: 1.67 (overlap) ^a^	Hb: 1.61 (overlap) ^a^	Hb: 1.64 (overlap) ^a^	Hb: 1.65 (overlap) ^a^	Hb: 1.68 (overlap) ^a^
6	4.56 (br d, 4.7)	4.36 (br d, 4.8)	4.48 (br d, 2.6)	4.48 (br d, 2.2)	4.66 (br d, 2.7)
10	Ha: 3.97 (d, 11.3)	Ha: 3.94 (d, 11.2)	1.15 (overlap)^a^	1.15 (overlap)^a^	Ha: 3.98 (d, 11.1)
	Hb: 3.73 (d, 11.3)	Hb: 3.69 (d, 11.2)			Hb: 3.72 (d, 11.1)
11	1.33 (s)	1.24 (s)	1.15 (overlap)^a^	1.15 (overlap)^a^	1.41 (s)
1′	Ha: 4.23 (d, 17.2)	4.97 (br t, 7.4)	3.74 (t, 7.0)	3.51 (t, 7.0)	
	Hb: 4.18 (d, 17.2)				
2′		3.49 (br d, 8.0)	2.58 (t, 7.1)	1.61 (m)	
3′				1.36 (m)	
4′		7.26 (s)	3.64 (s)	1.66 (m)	
5′				2.89 (t, 7.7)	
7′		8.71 (s)			

^a^ Signals might be interchangeable.

**Table 2:** The ^13^C NMR data (150 MHz) of **1–5** in CD_3_OD (*δ* in ppm).

Position	1	2	3	4	5
1	61.9, C	61.6, C	55.3, C	55.2, C	64.3, C
2	55.3, C	55.0, C	55.3, C	55.2, C	55.9, C
3	85.3, CH	85.3, CH	85.1, CH	85.2, CH	86.7, CH
4	24.6 CH_2_	24.5, CH_2_	24.6, CH_2_	24.6, CH_2_	24.7, CH_2_
5	25.1, CH_2_	24.9, CH_2_	24.6, CH_2_	24.6, CH_2_	24.6, CH_2_
6	83.2, CH	83.2, CH	85.1, CH	85.2, CH	84.2, CH
7	180.9, C	180.7, C	183.1, C	183.6, C	176.0, C
9	182.5, C	182.7, C	183.1, C	183.6, C	177.9, C
10	59.5, CH_2_	59.4, CH_2_	12.6, CH_3_	12.5, CH_3_	59.1, CH_2_
11	12.2, CH_3_	12.2, CH_3_	12.6, CH_3_	12.5, CH_3_	12.7, CH_3_
1′	40.5, CH_2_	53.2, CH	35.8, CH_2_	39.4, CH_2_	
2′	169.7, C	25.1, CH_2_	32.7, CH_2_	27.89, CH_2_	
3′		130.8, C	172.9, C	24.3, CH_2_	
4′		119.1, CH	52.4, CH_3_	27.9, CH_2_	
5′				40.5, CH_2_	
6′		134.7, CH			
8′		171.1, C			

## Data Availability

All the data in this research are presented in manuscript and supplementary material.

## Supplementary Materials

The following supporting information can be downloaded at: https://www.mdpi.com/article/10.3390/molecules28062822/s1, Figures S1–S6: NMR spectra of **1**. Figure S7: HRESIMS of **1**. Figure S8: CD spectrum of **1**. Figures S9–S14: NMR spectra of **2**. Figure S15: HRESIMS of **2**. Figure S16: CD spectrum of **2**. Figures S17–S22: NMR spectra of **3**. Figure S23: HRESIMS of **3**. Figure S24: CD spectrum of **3**. Figures S25–S30: NMR spectra of **4**. Figure S31: HRESIMS of **4**. Figure S32: CD spectrum of **4**. Figures S33–S38: NMR spectra of **5**. Figure S39: HRESIMS of **5**. Figure S40: CD spectrum of **5**. Scheme S1: Semi-synthesis of compounds **2a** and **2b**. Figures S41–S42: NMR spectra of **2a**. Figures S43–S44: NMR spectra of **2b**. Figure S45: HPLC analyses of compounds **2**, **2a**, and **2b**. Figures S46–S50: Optimized geometries of predominant conformers for **1–5**. Tables S1–S5: The cartesian coordinates of the lowest energy conformers for **1–5**.

## Sample Availability

Samples of compounds **1–8** are available from the authors.
