# Supplementary material for: Five New Cantharidin Derivatives from the Insect Mylabris cichorii L. and Their Potential against Kidney Fibrosis In Vitro
Source: Molecules. 2023 Mar 21;28(6):2822. doi: 10.3390/molecules28062822 (PMC10056085; doi:10.3390/molecules28062822)
Supplement: Supplementary file 1 [file molecules-28-02822-s001.zip › molecules-2268529-supplementary.pdf]

Supplementary Materials

Five New Cantharidin Derivatives from the Insect *Mylabris cichorii* L. and Their Potential against Kidney Fibrosis In Vitro

Ke-Ming Li [1,2], Ji-Jun Li [2], Li Wan [1,*] and Yong-Xian Cheng [1,2,*,†]

1 School of Pharmacy, Chengdu University of Traditional Chinese Medicine, Chengdu 611137, China
2 Institute for Inheritance-Based Innovation of Chinese Medicine, Medical School of Pharmaceutical Sciences, Shenzhen University, Shenzhen 518060, China
* Correspondence: wanli@cdutcm.edu.cn (L.W.); yxcheng@szu.edu.cn (Y.-X.C.); Tel./Fax: +86-0755-26902073 (Y.-X.C.)
† Lead contact.

Contents

Supplementary Figures

Figure S1. ^{1}H NMR spectrum of **1** in CD_3OD
Figure S2. ^{13}C NMR and DEPT spectra of **1** in CD_3OD
Figure S3. HSQC spectrum of **1** in CD_3OD
Figure S4. HMBC spectrum of **1** in CD_3OD
Figure S5. ^{1}H-^{1}H COSY spectrum of **1** in CD_3OD
Figure S6. ROESY spectrum of **1** in CD_3OD
Figure S7. HRESIMS of **1**
Figure S8. CD spectrum of **1**
Figure S9. ^{1}H NMR spectrum of **2** in CD_3OD
Figure S10. ^{13}C NMR and DEPT spectra of **2** in CD_3OD
Figure S11. HSQC spectrum of **2** in CD_3OD
Figure S12. HMBC spectrum of **2** in CD_3OD
Figure S13. ^{1}H-^{1}H COSY spectrum of **2** in CD_3OD
Figure S14. ROESY spectrum of **2** in CD_3OD
Figure S15. HRESIMS of **2**
Figure S16. CD spectrum of **2**
Figure S17. ^{1}H NMR spectrum of **3** in CD_3OD
Figure S18. ^{13}C NMR and DEPT spectra of **3** in CD_3OD
Figure S19. HSQC spectrum of **3** in CD_3OD
Figure S20. HMBC spectrum of **3** in CD_3OD
Figure S21. ^{1}H-^{1}H COSY spectrum of **3** in CD_3OD
Figure S22. ROESY spectrum of **3** in CD_3OD
Figure S23. HRESIMS of **3**
Figure S24. CD spectrum of **3**
Figure S25. ^{1}H NMR spectrum of **4** in CD_3OD
Figure S26. ^{13}C NMR and DEPT spectra of **4** in CD_3OD
Figure S27. HSQC spectrum of **4** in CD_3OD
Figure S28. HMBC spectrum of **4** in CD_3OD
Figure S29. ^{1}H-^{1}H COSY spectrum of **4** in CD_3OD
Figure S30. ROESY spectrum of **4** in CD_3OD
Figure S31. HRESIMS of **4**
Figure S32. CD spectrum of **4**
Figure S33. ^{1}H NMR spectrum of **5** in CD_3OD
Figure S34. ^{13}C NMR and DEPT spectra of **5** in CD_3OD
Figure S35. HSQC spectrum of **5** in CD_3OD
Figure S36. HMBC spectrum of **5** in CD_3OD
Figure S37. ^{1}H-^{1}H COSY spectrum of **5** in CD_3OD
Figure S38. ROESY spectrum of **5** in CD_3OD
Figure S39. HRESIMS of **5**
Figure S40. CD spectrum of **5**

Scheme S1. Semi-synthesis of compounds **2a** and **2b**
Figure S41. ^{1}H NMR spectrum of **2a** in CD_3OD
Figure S42. ^{13}C NMR spectrum of **2a** in CD_3OD
Figure S43. ^{1}H NMR spectrum of **2b** in CD_3OD
Figure S44. ^{13}C NMR spectrum of **2b** in CD_3OD
Figure S45. HPLC analyses of compounds **2**, **2a** and 2**b**
Figures S46-50. Optimized geometries of predominant conformers for **1**-**5**
Tables S1–S5. The Cartesian coordinates of the lowest energy conformers for **1**-**5**

Supplementary Figures

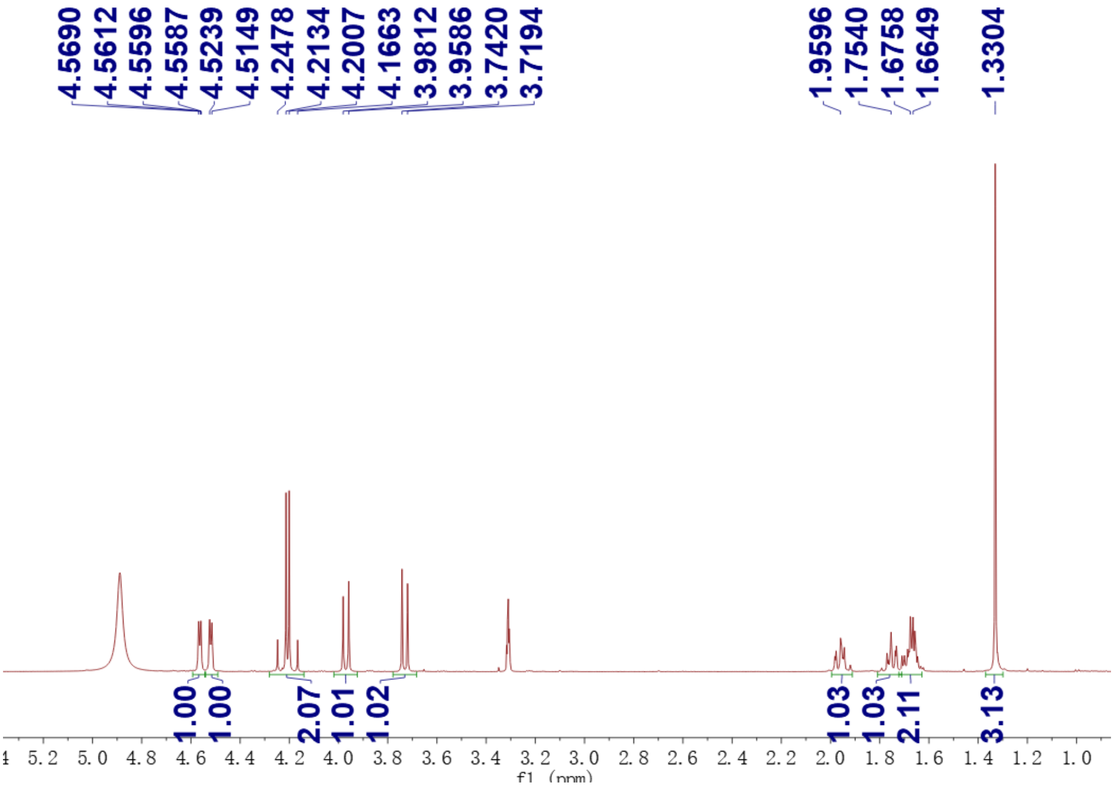

Figure S1. ^{1}H NMR spectrum of **1** in CD_3OD

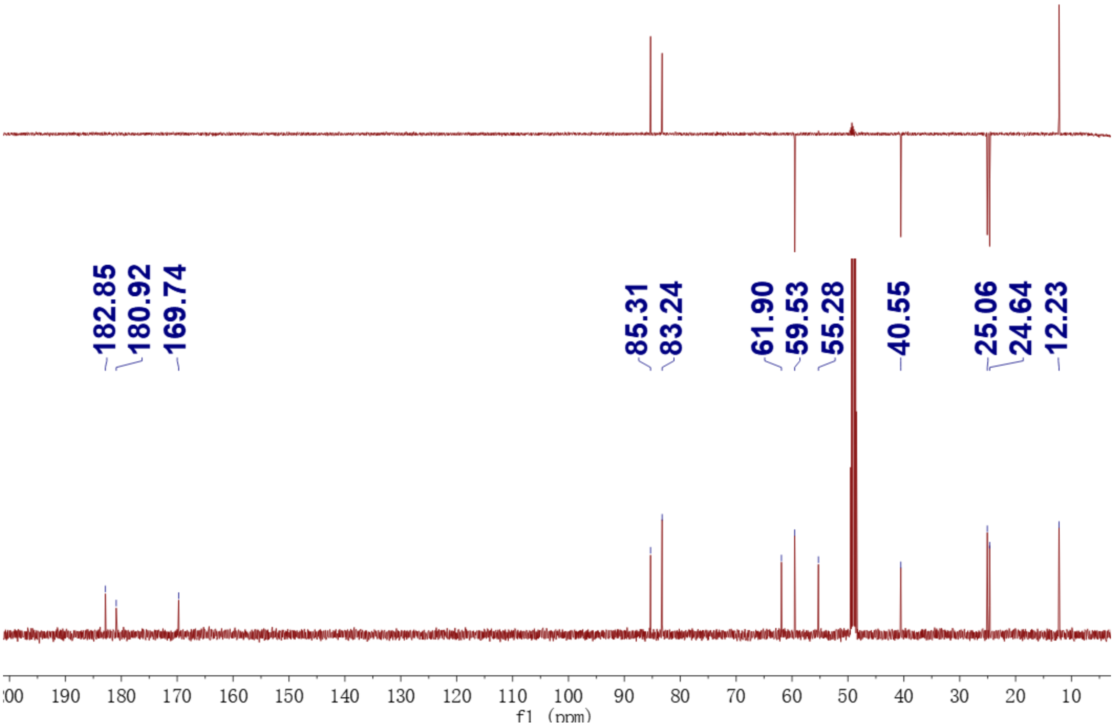

Figure S2. ^{13}C NMR and DEPT spectra of **1** in CD_3OD

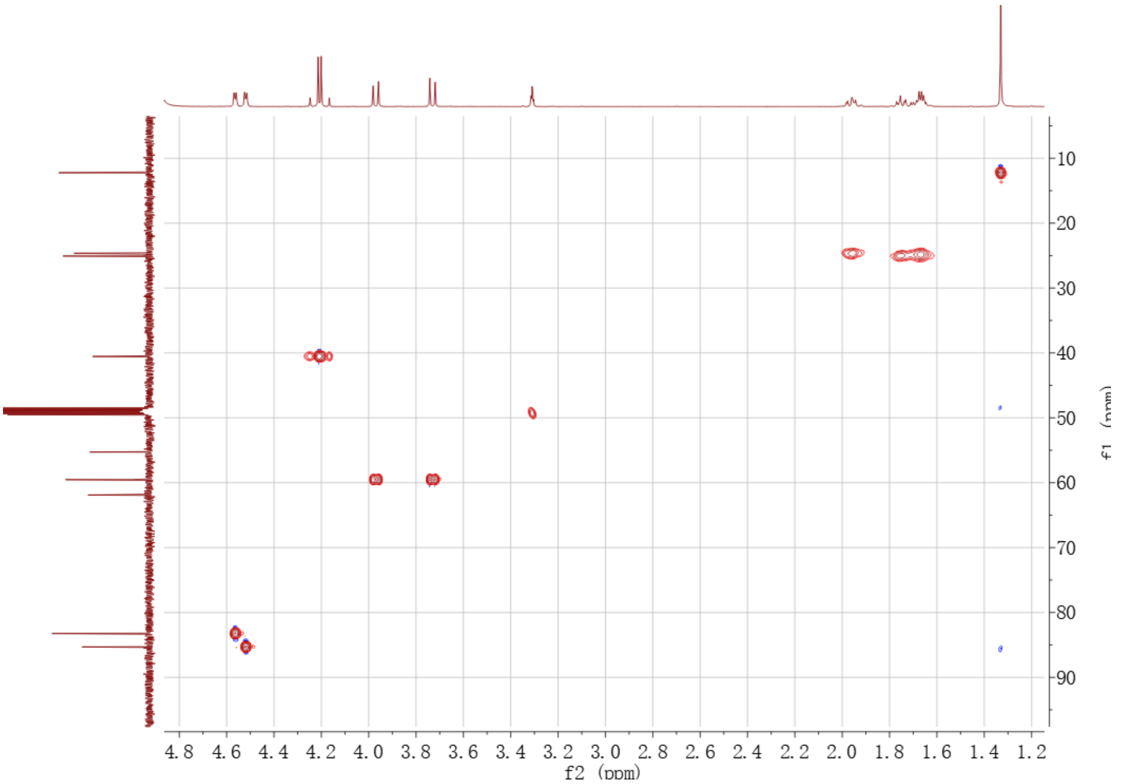

Figure S3. HSQC spectrum of **1** in CD_3OD

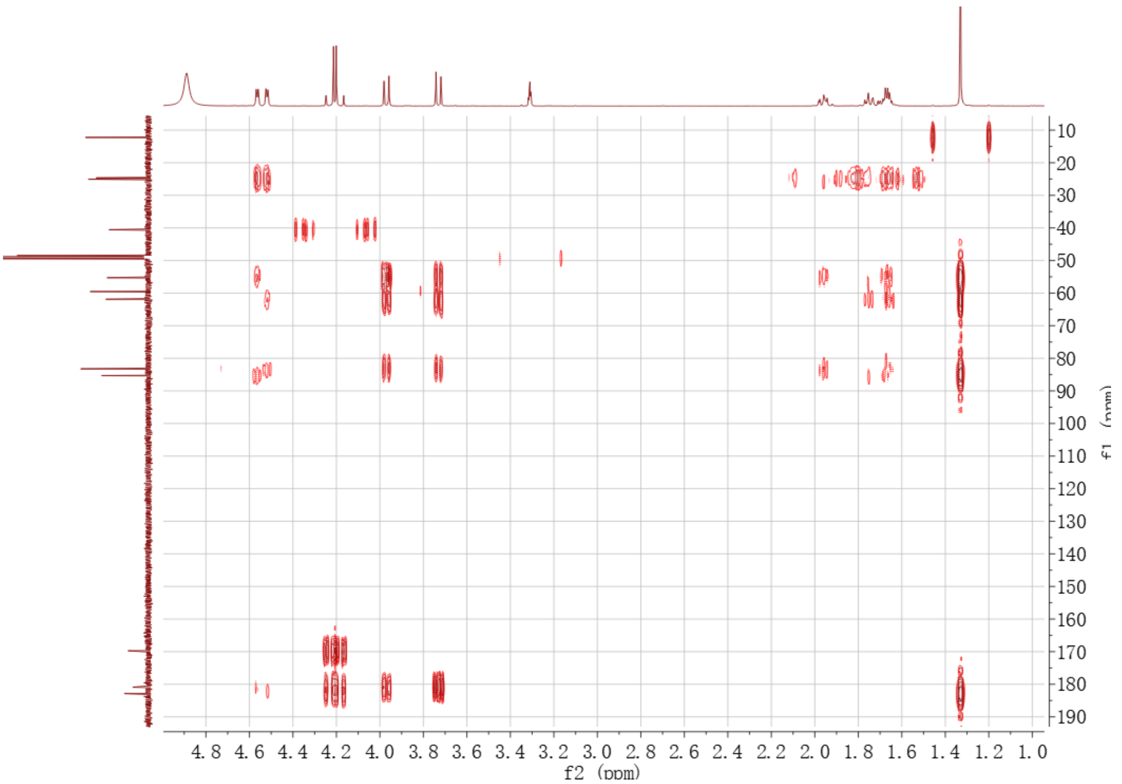

Figure S4. HMBC spectrum of **1** in CD_3OD

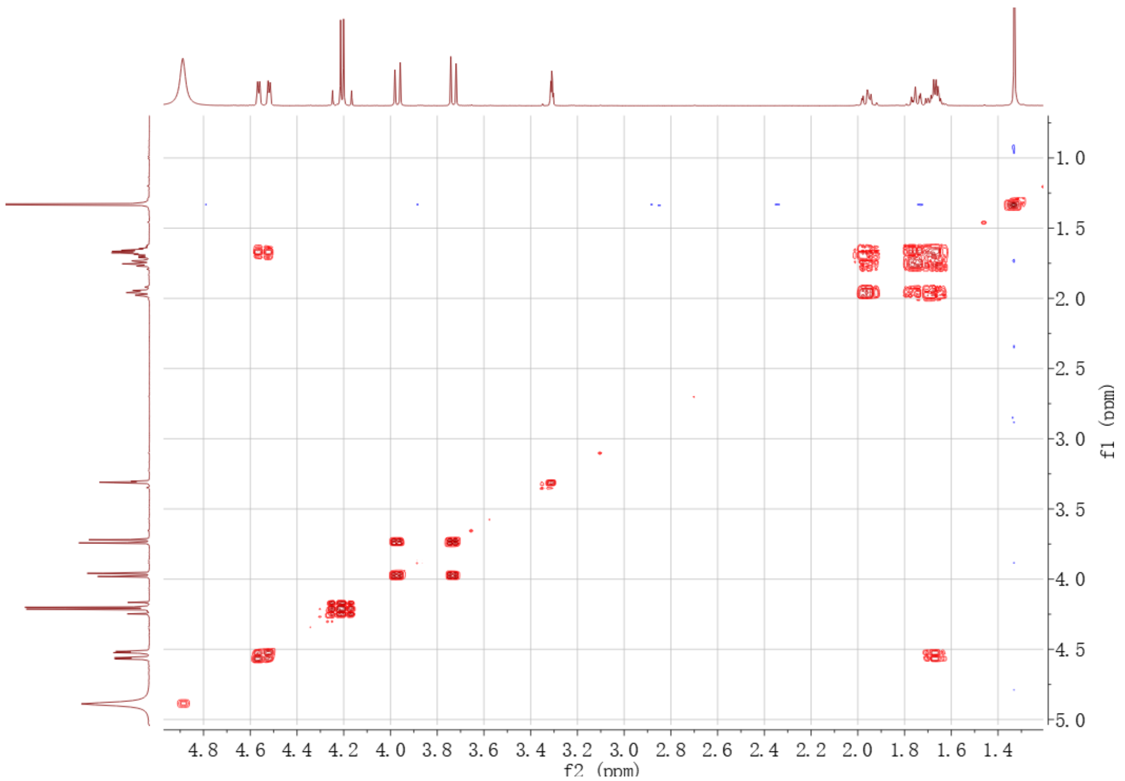

Figure S5. ^{1}H-^{1}H COSY spectrum of **1** in CD_3OD

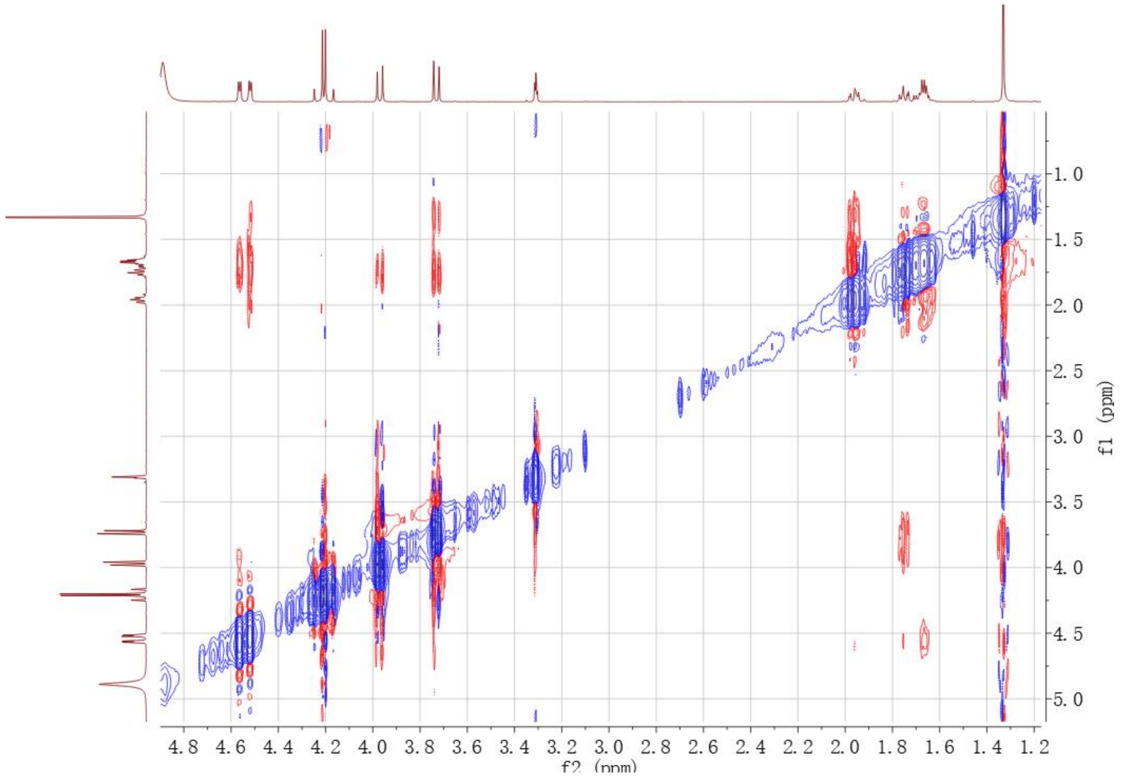

Figure S6. ROESY spectrum of **1** in CD_3OD

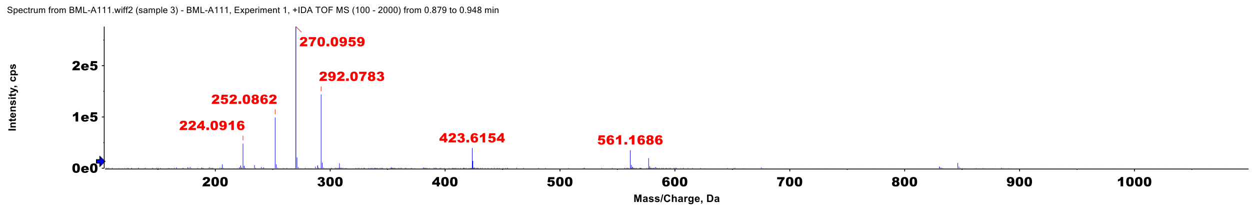

Hit	Formula	m/z	RDB	ppm	MS Rank	Found
1	C12H15NO6	270.0972	6.0	-4.9	1	NA/NA

Figure S7. HRESIMS of **1**

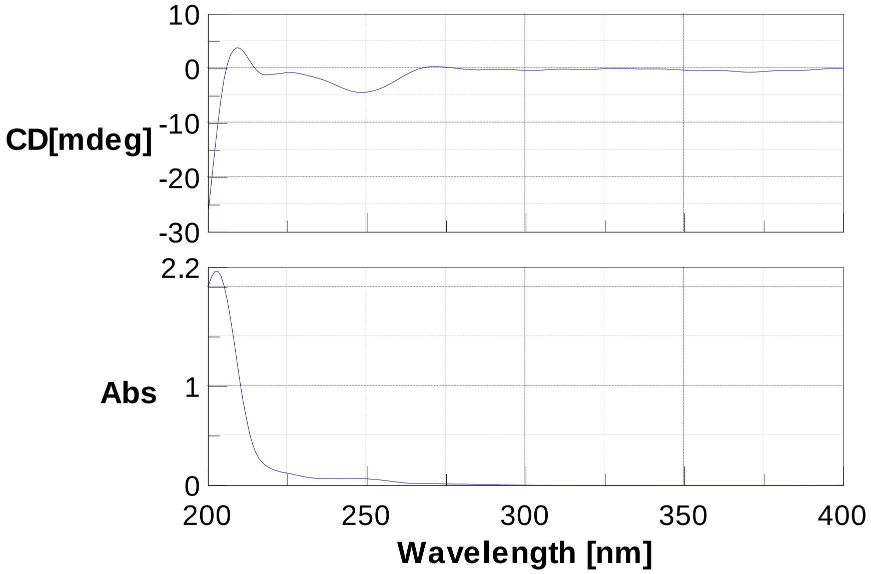

Figure S8. CD spectrum of **1**

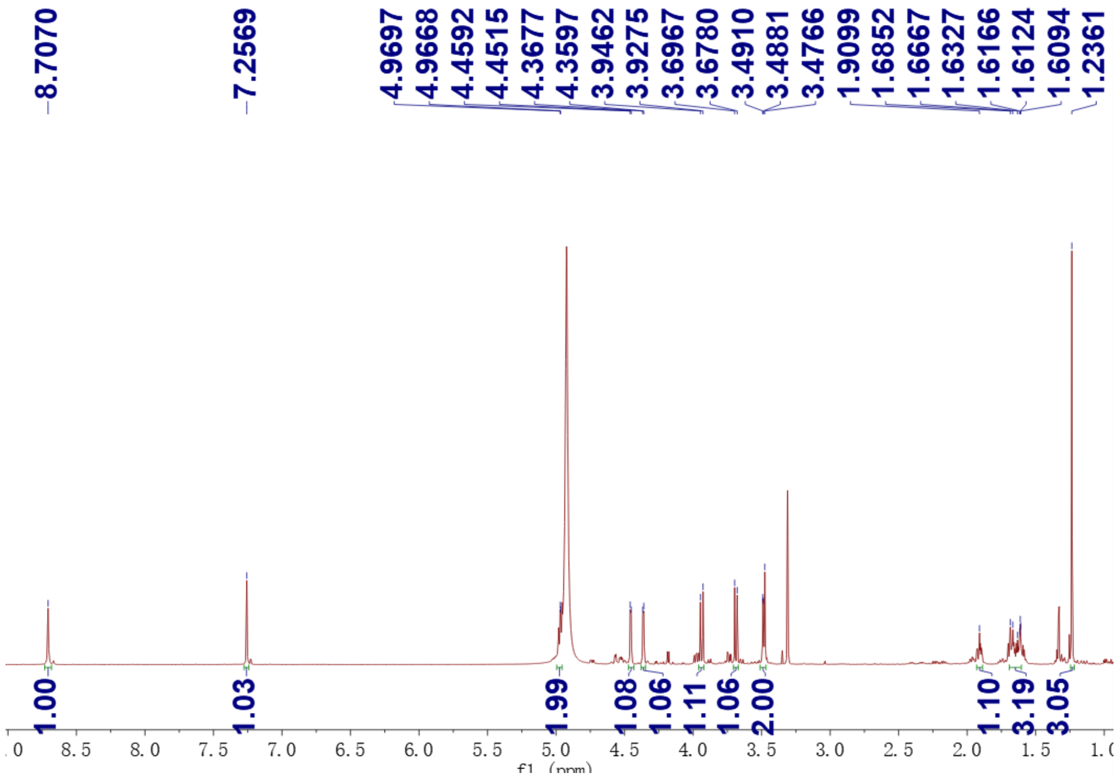

Figure S9. ^{1}H NMR spectrum of **2** in CD_3OD

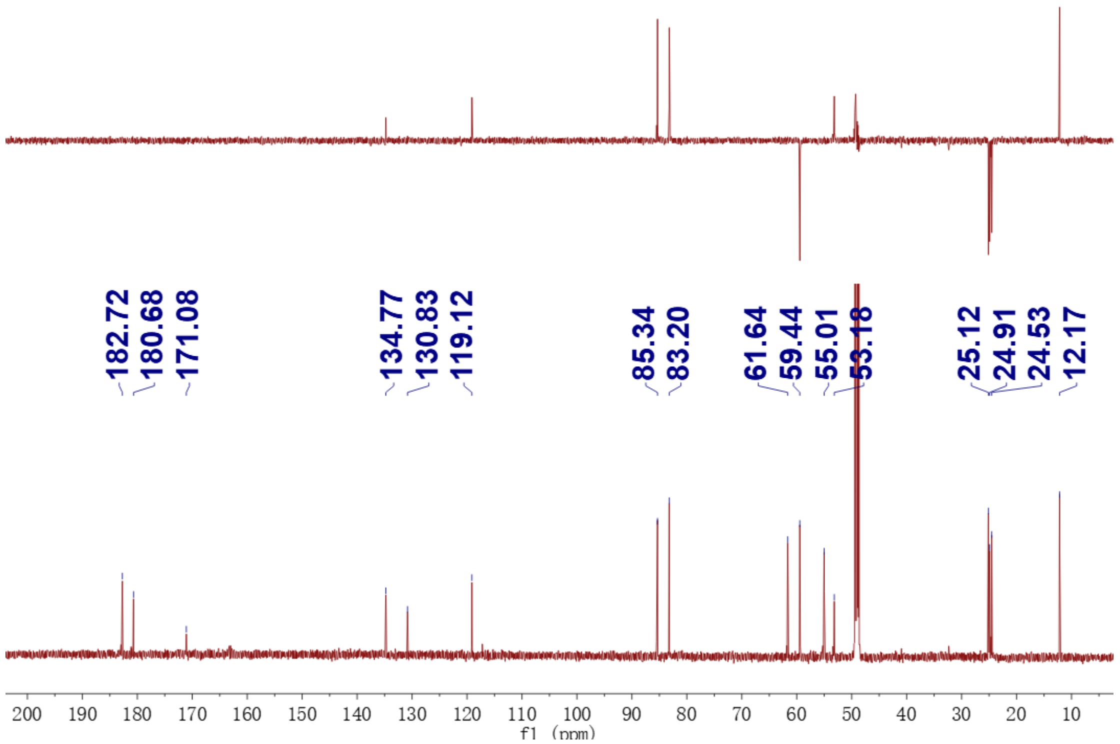

Figure S10. ^{13}C NMR and DEPT spectra of **2** in CD_3OD

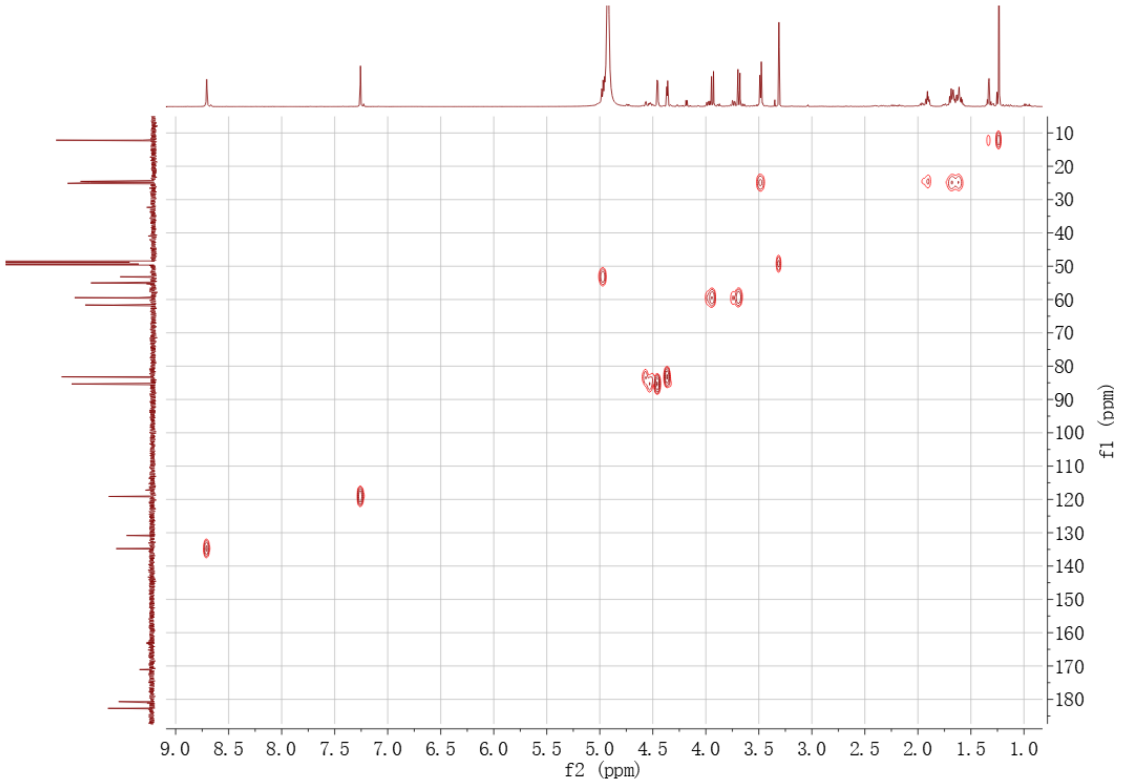

Figure S11. HSQC spectrum of **2** in CD_3OD

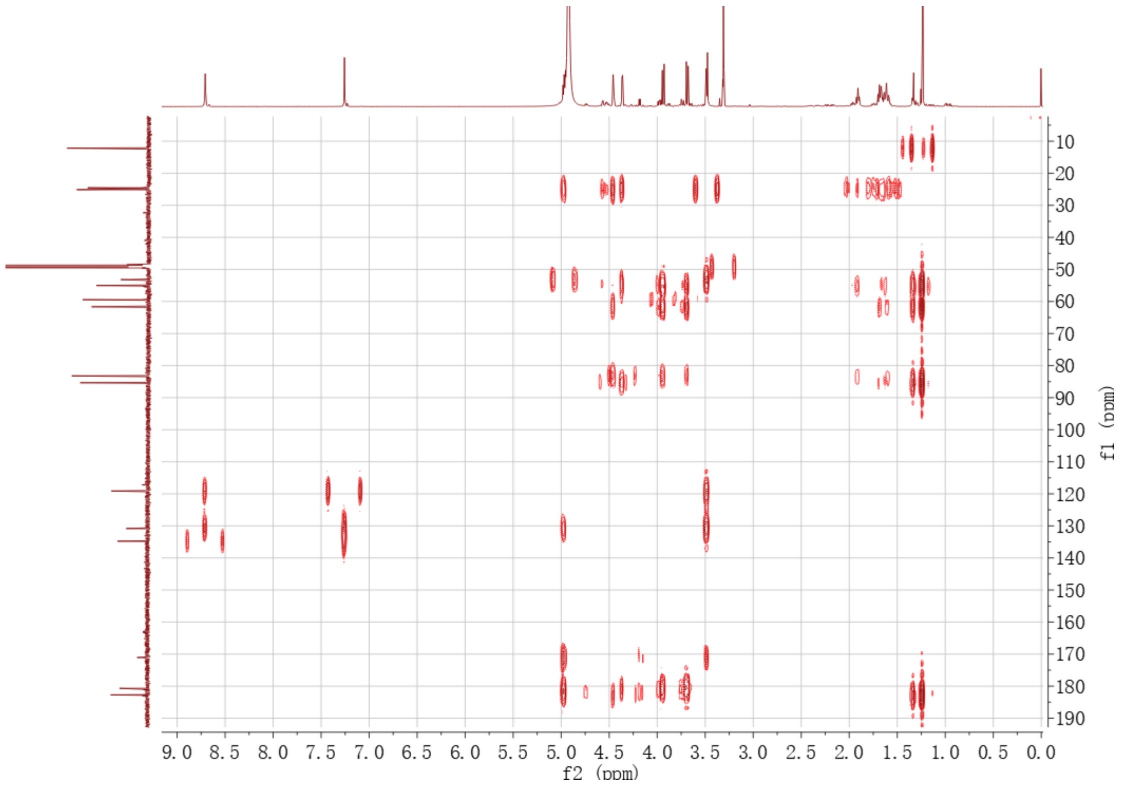

Figure S12. HMBC spectrum of **2** in CD_3OD

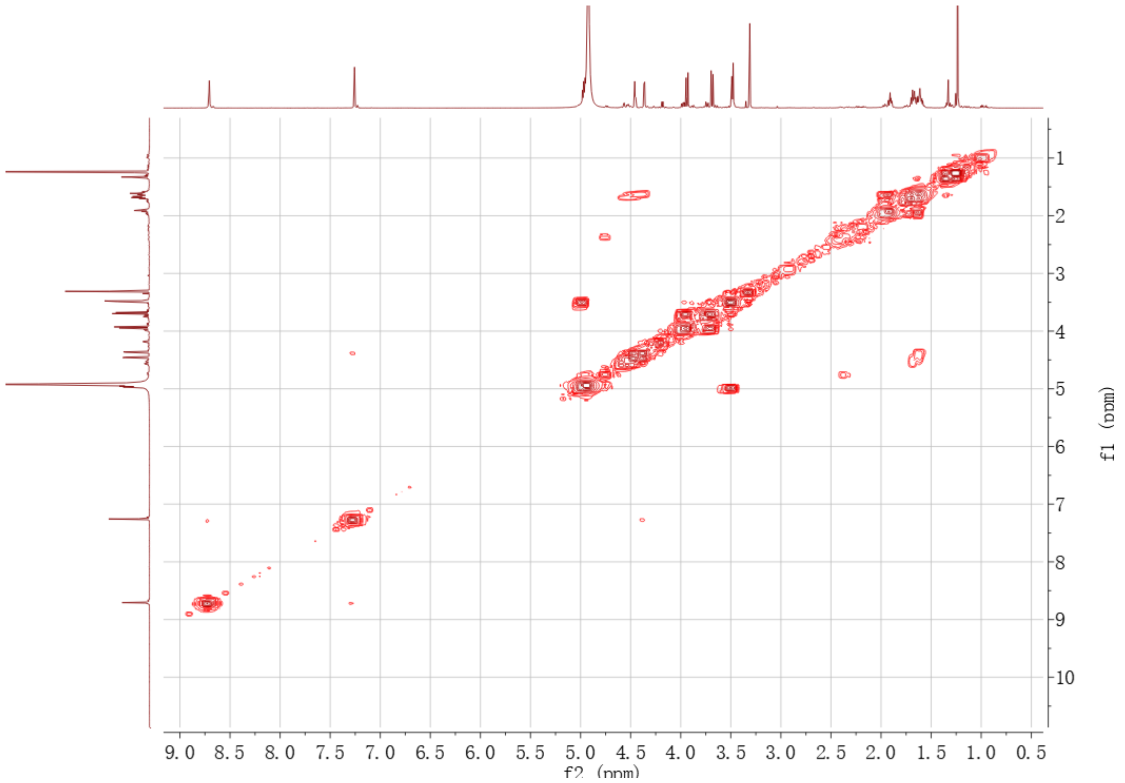

Figure S13. ^{1}H-^{1}H COSY spectrum of **2** in CD_3OD

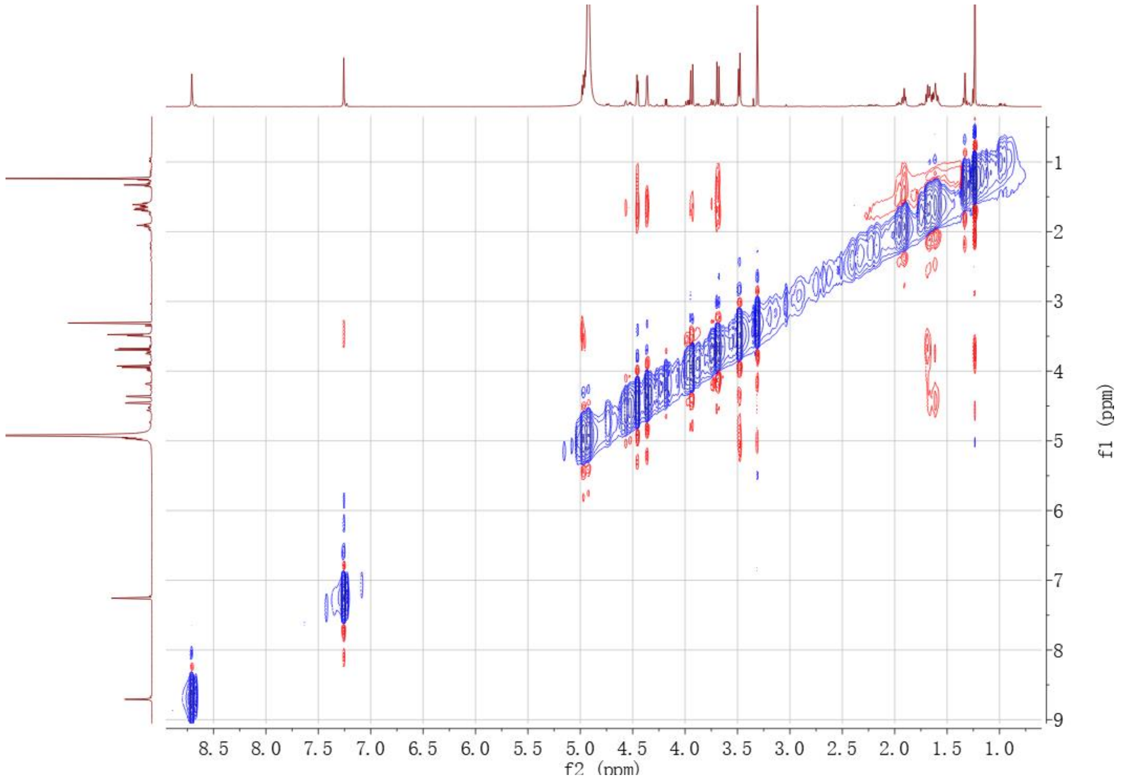

Figure S14. ROESY spectrum of **2** in CD_3OD

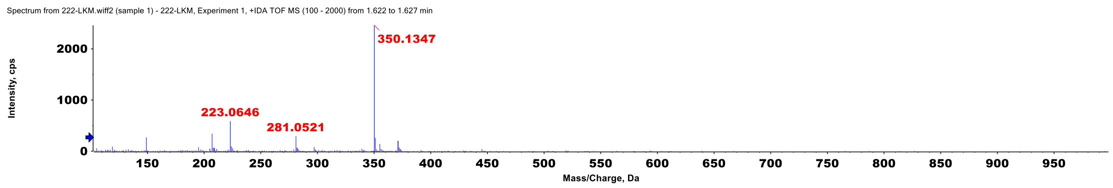

Hit	Formula	m/z	RDB	ppm	MS Rank	Found
1	C18H22O7	350.1360	8.5	-3.7	1	NA/NA

Figure S15. HRESIMS of **2**

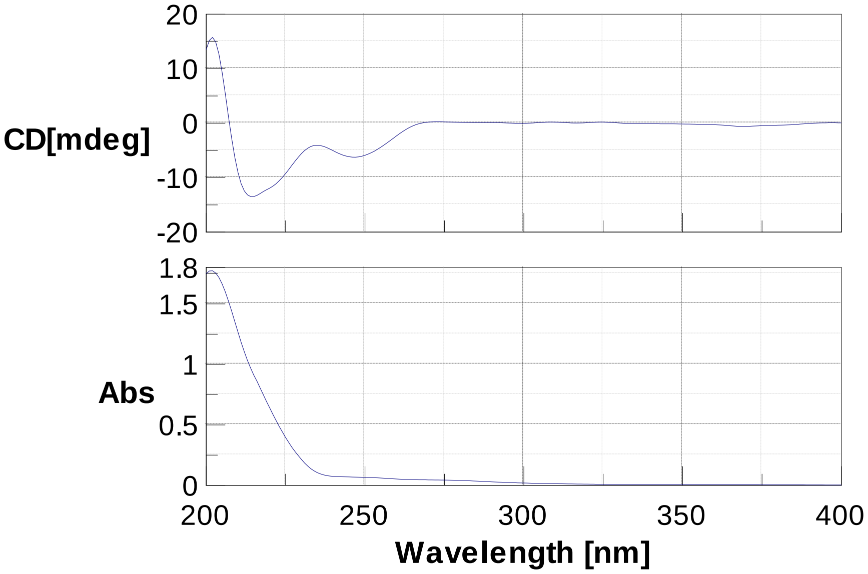

Figure S16. CD spectrum of **2**

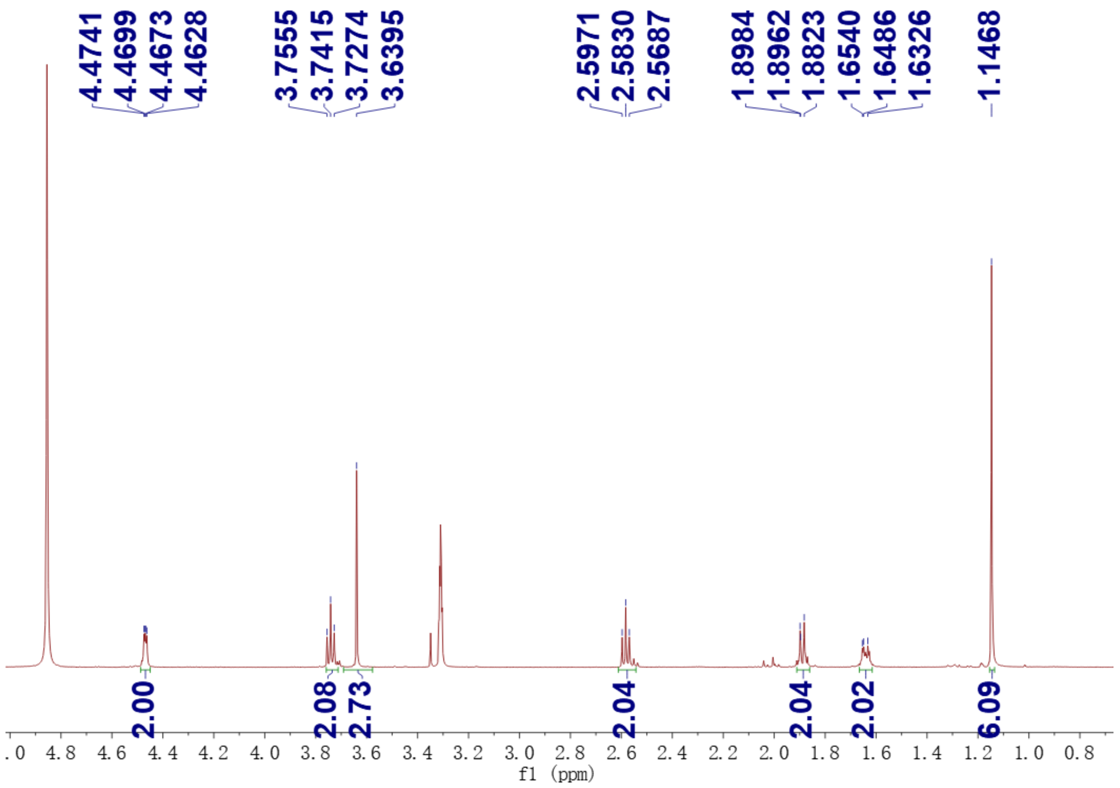

Figure S17. ^{1}H NMR spectrum of **3** in CD_3OD

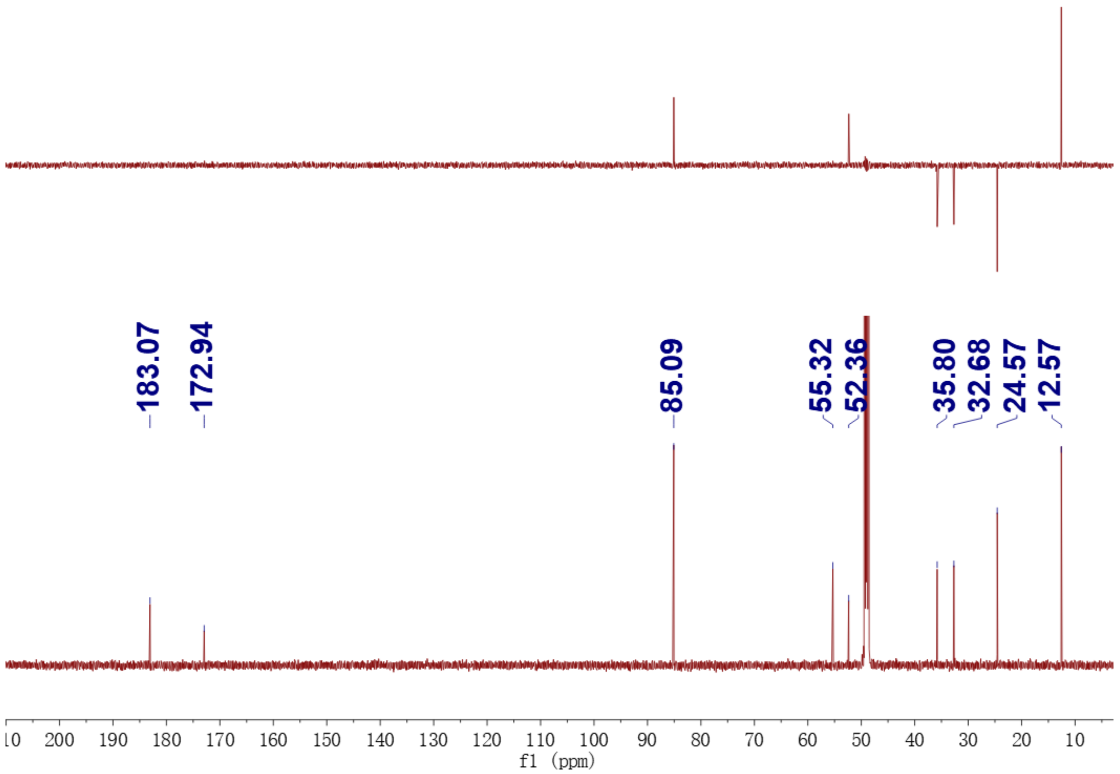

Figure S18. ^{13}C NMR and DEPT spectra of **3** in CD_3OD

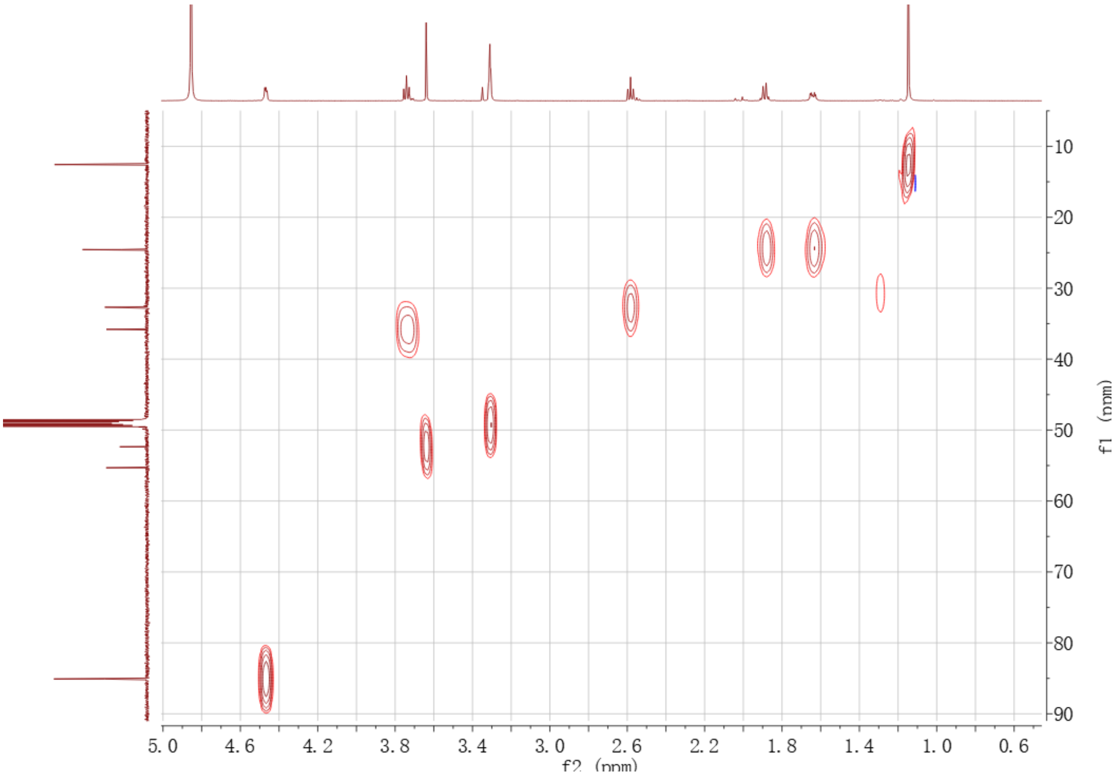

Figure S19. HSQC spectrum of **3** in CD_3OD

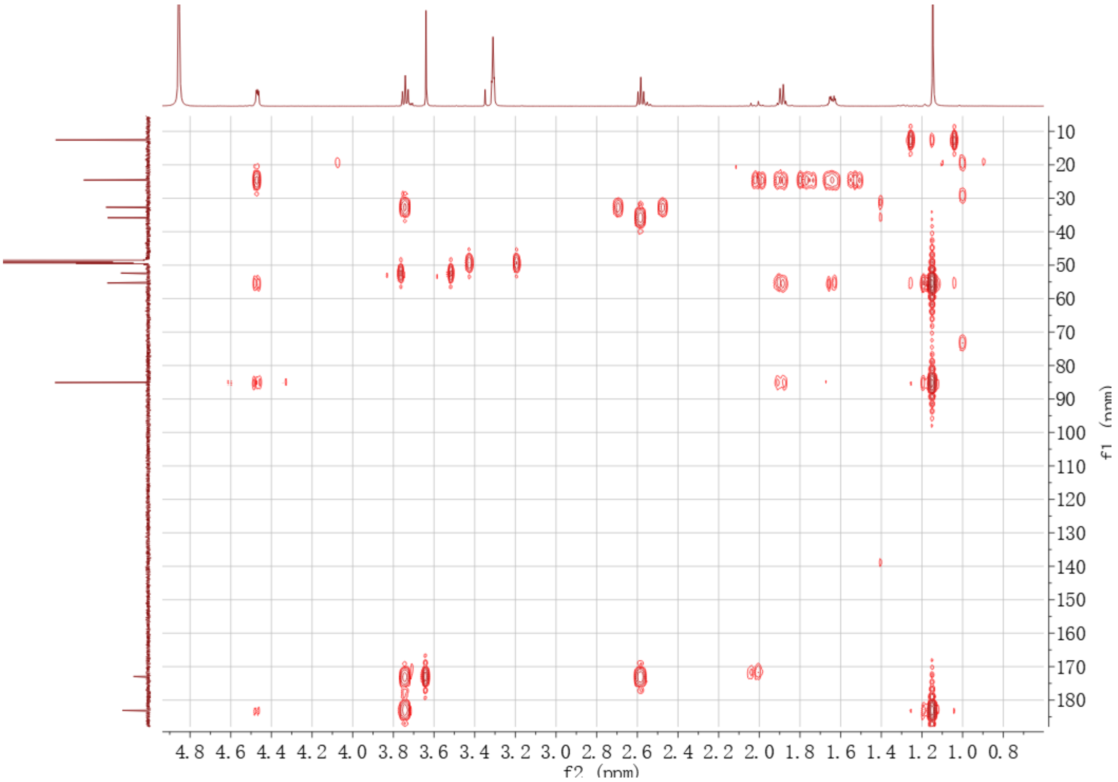

Figure S20. HMBC spectrum of **3** in CD_3OD

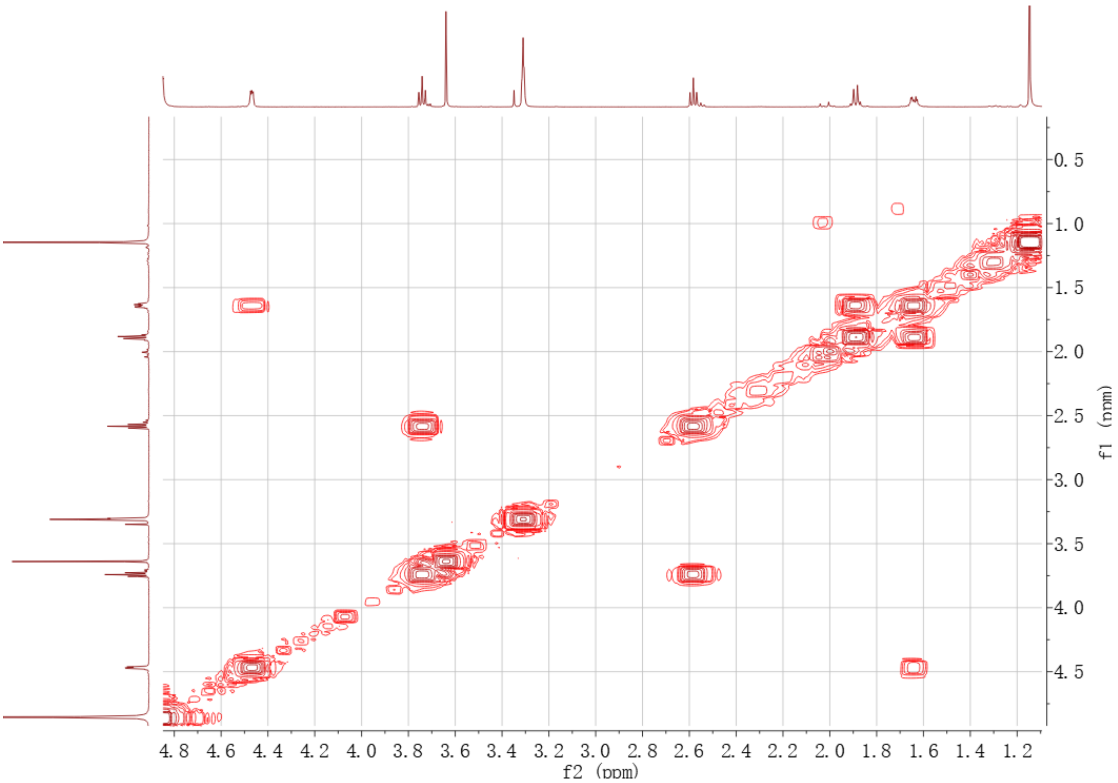

Figure S21. ^1H-^1H COSY spectrum of **3** in CD_3OD

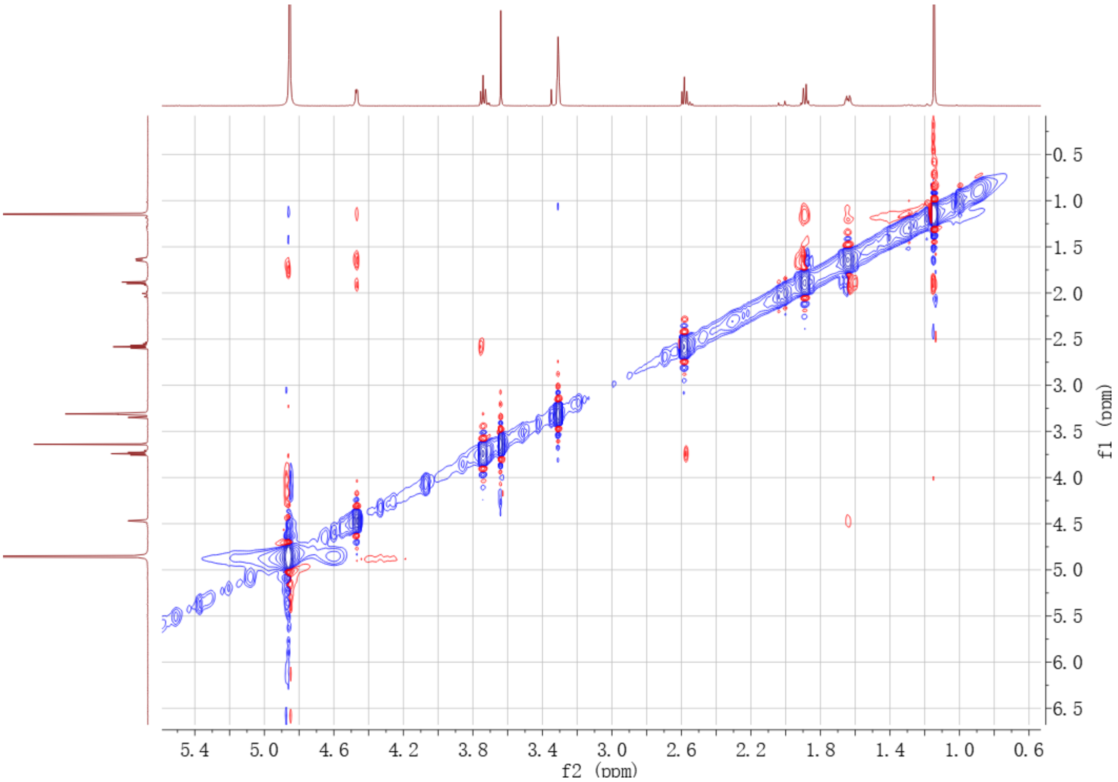

Figure S22. ROESY spectrum of **3** in CD_3OD

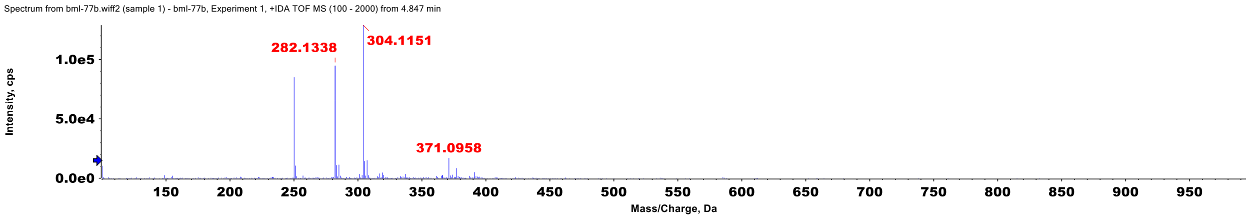

Hit	Formula	m/z	RDB	ppm	MS Rank	Found
1	C14H19NO5	282.1336	6.0	0.7	1	NA/NA

Figure S23. HRESIMS of **3**

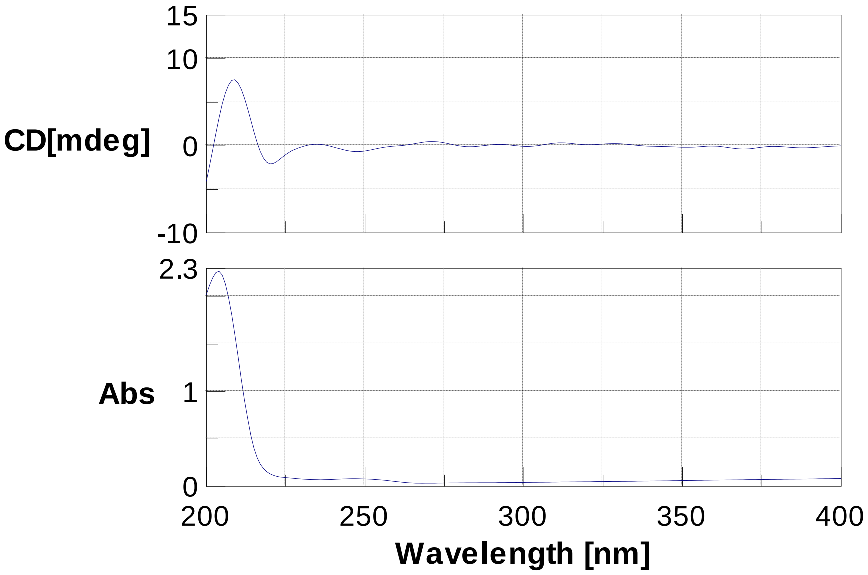

Figure S24. CD spectrum of **3**

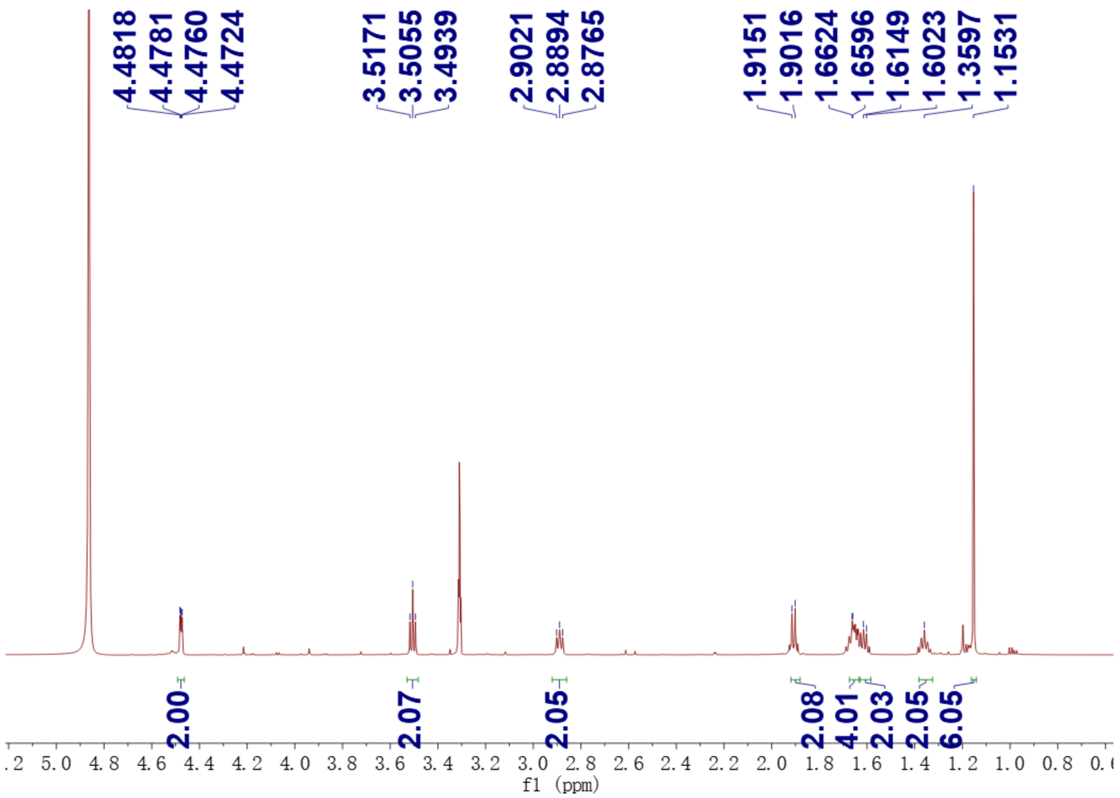

Figure S25. ^{1}H NMR spectrum of **4** in CD_3OD

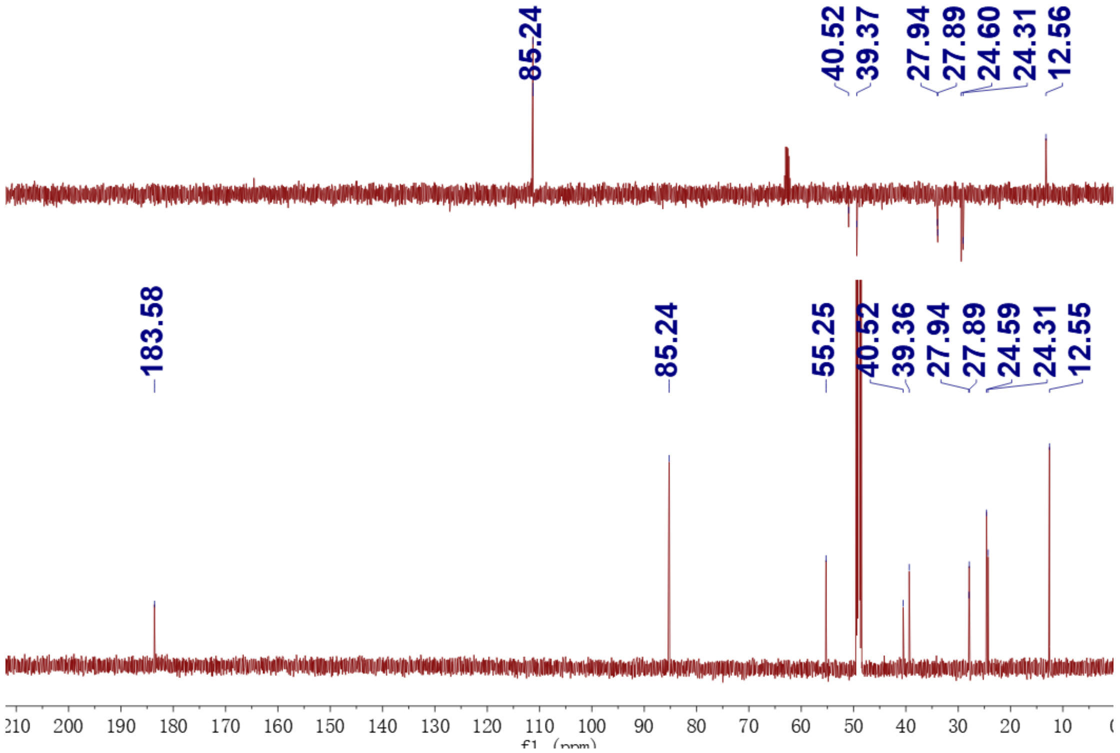

Figure S26. ^{13}C NMR and DEPT spectra of **4** in CD_3OD

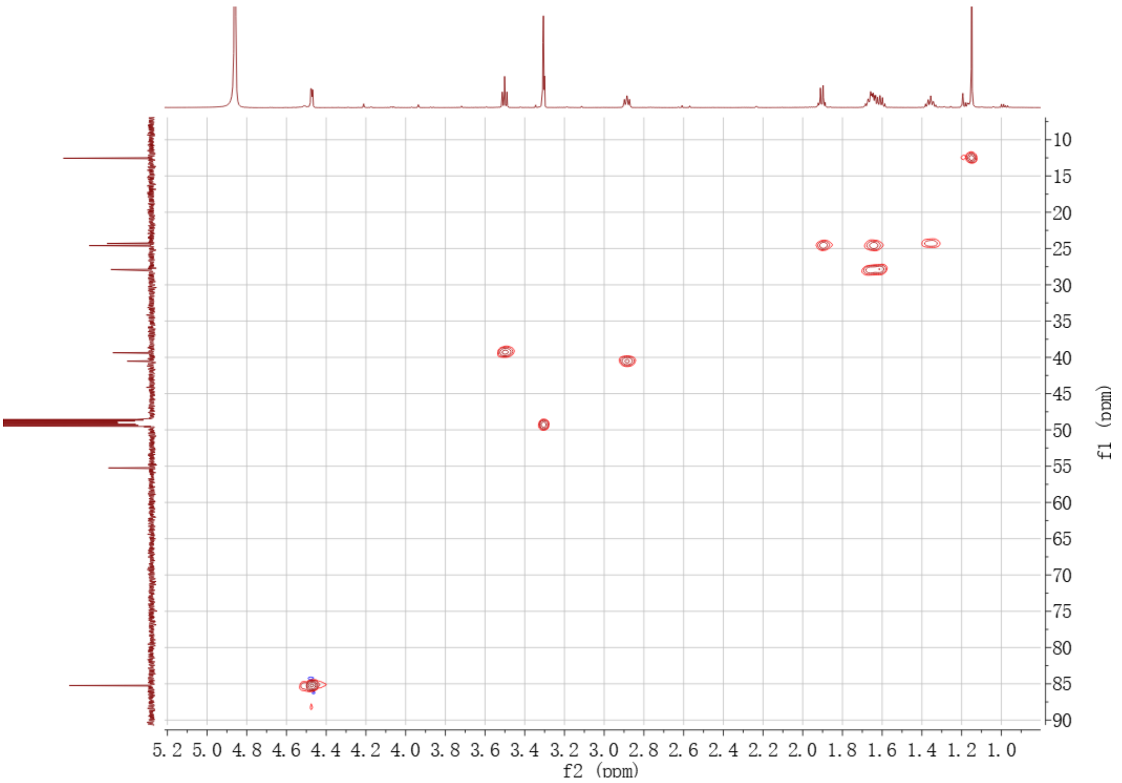

Figure S27. HSQC spectrum of **4** in CD_3OD

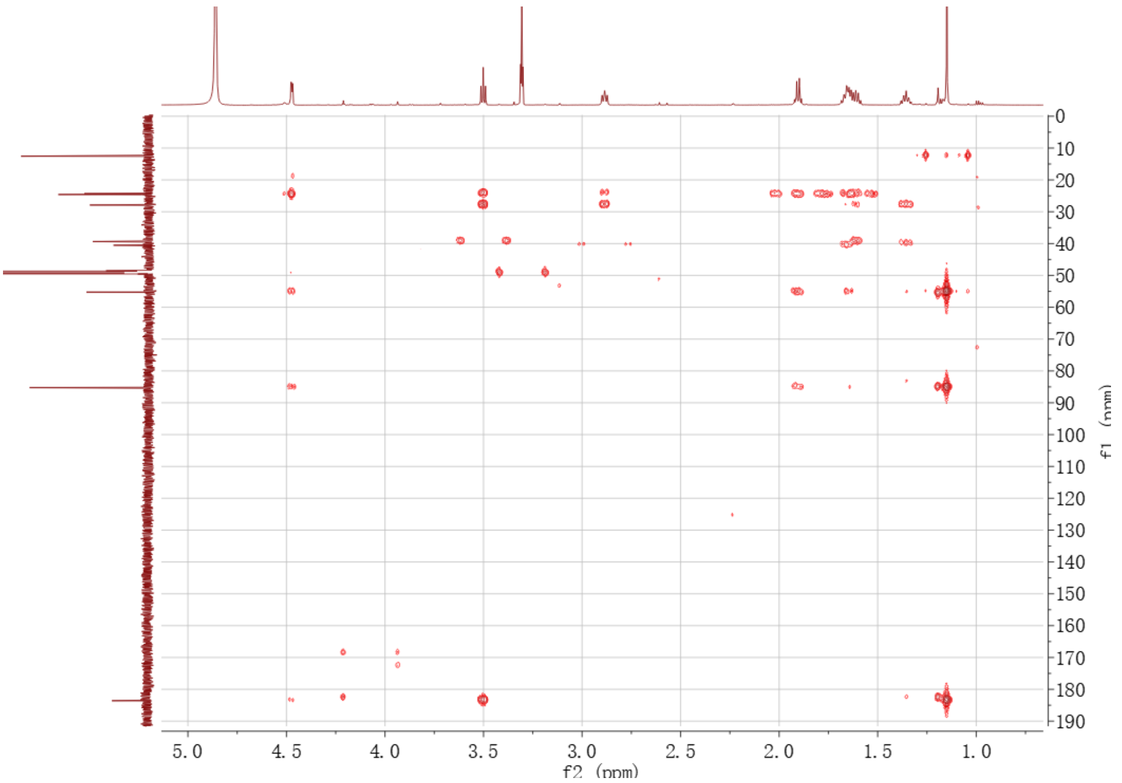

Figure S28. HMBC spectrum of **4** in CD_3OD

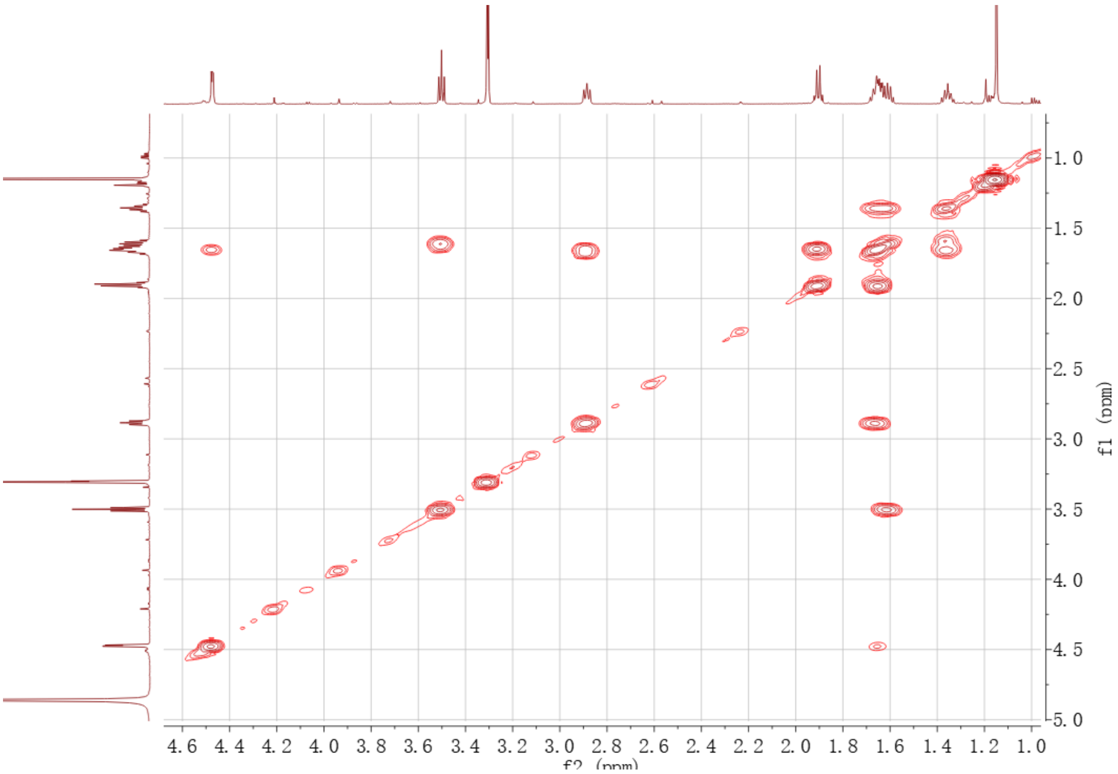

Figure S29. ^{1}H-^{1}H COSY spectrum of **4** in CD_3OD

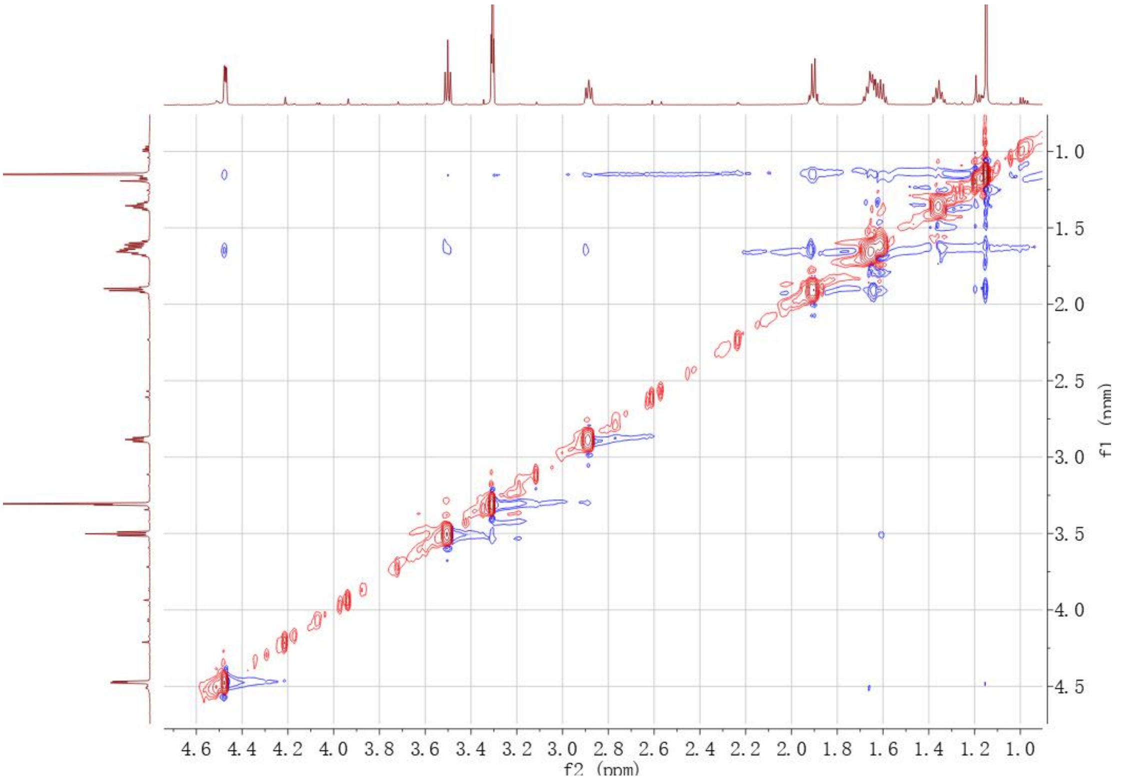

Figure S30. ROESY spectrum of **4** in CD_3OD

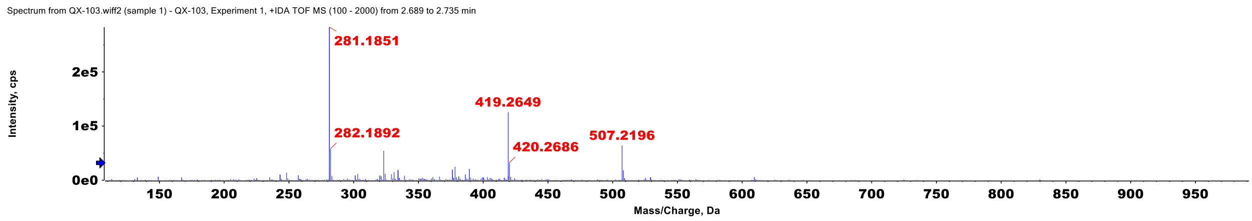

Hit	Formula	m/z	RDB	ppm	MS Rank	Found
1	C15H24N2O3	281.1860	5.0	-3.1	1	NA/NA

Figure S31. HRESIMS of **4**

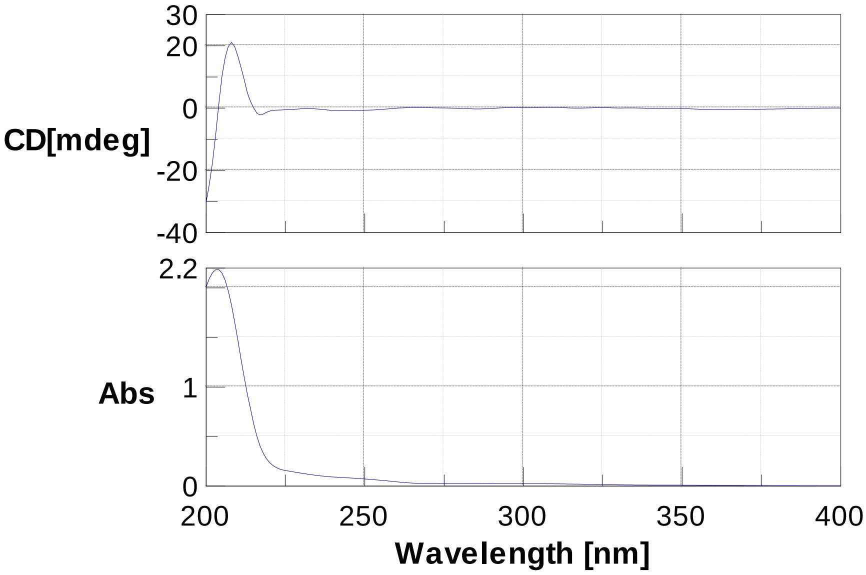

Figure S32. CD spectrum of **4**

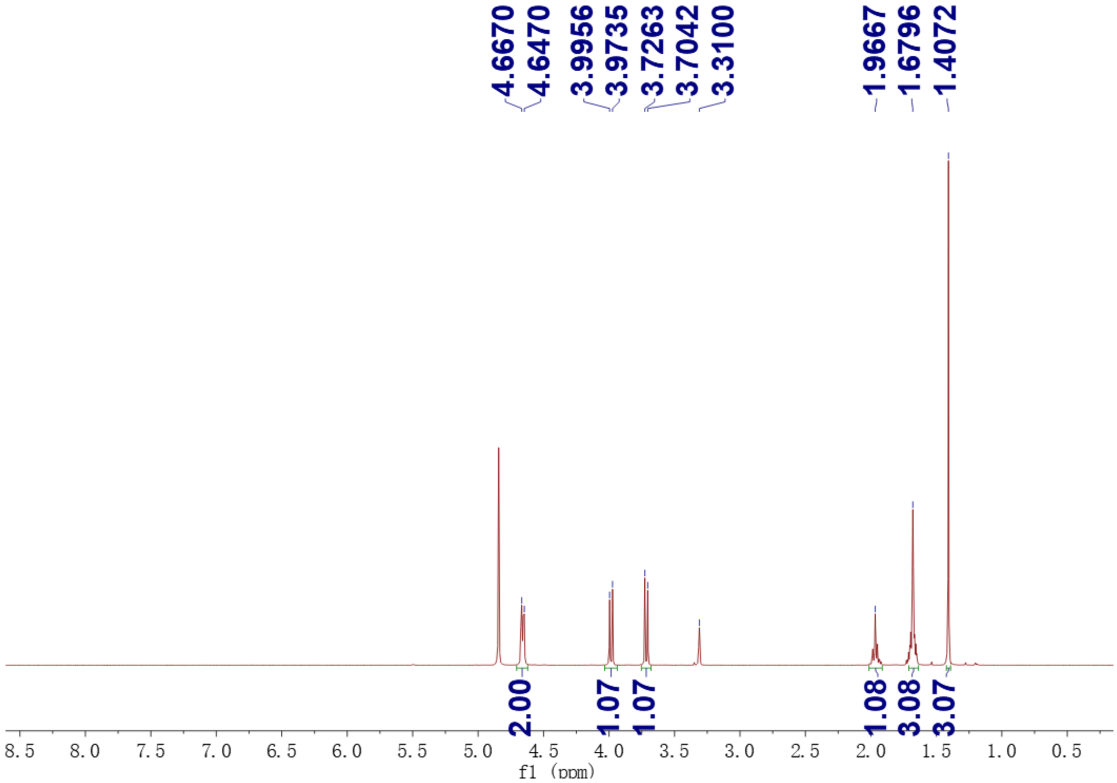

Figure S33. ^{1}H NMR spectrum of **5** in CD_3OD

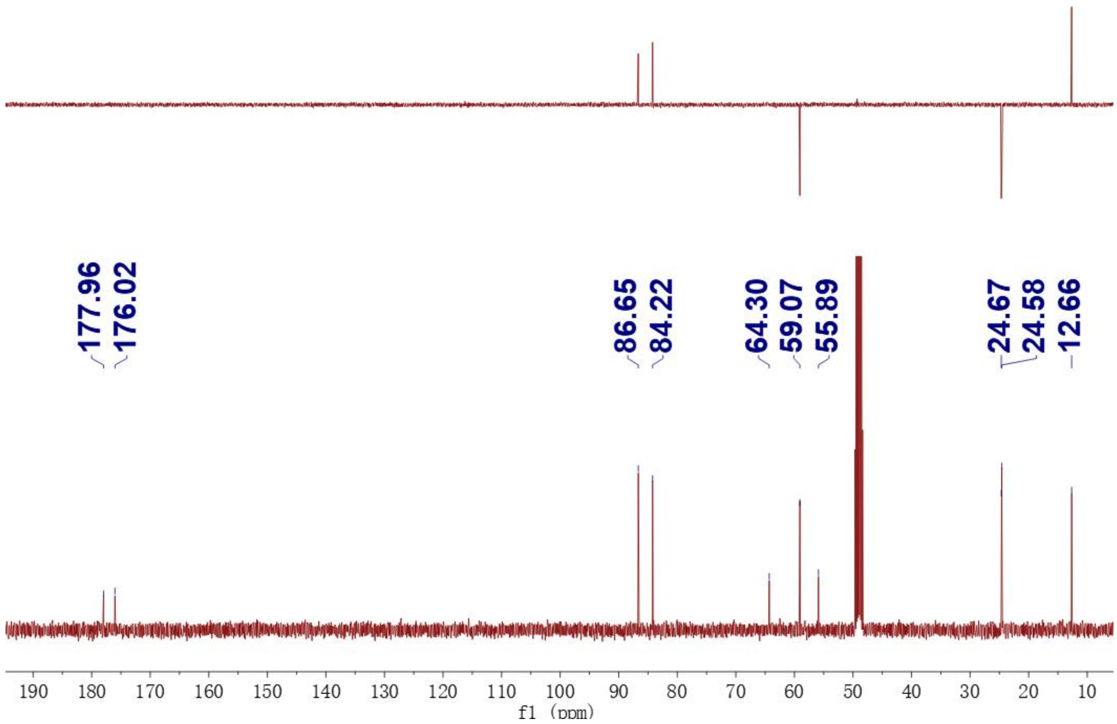

Figure S34. ^{13}C NMR and DEPT spectra of **5** in CD_3OD

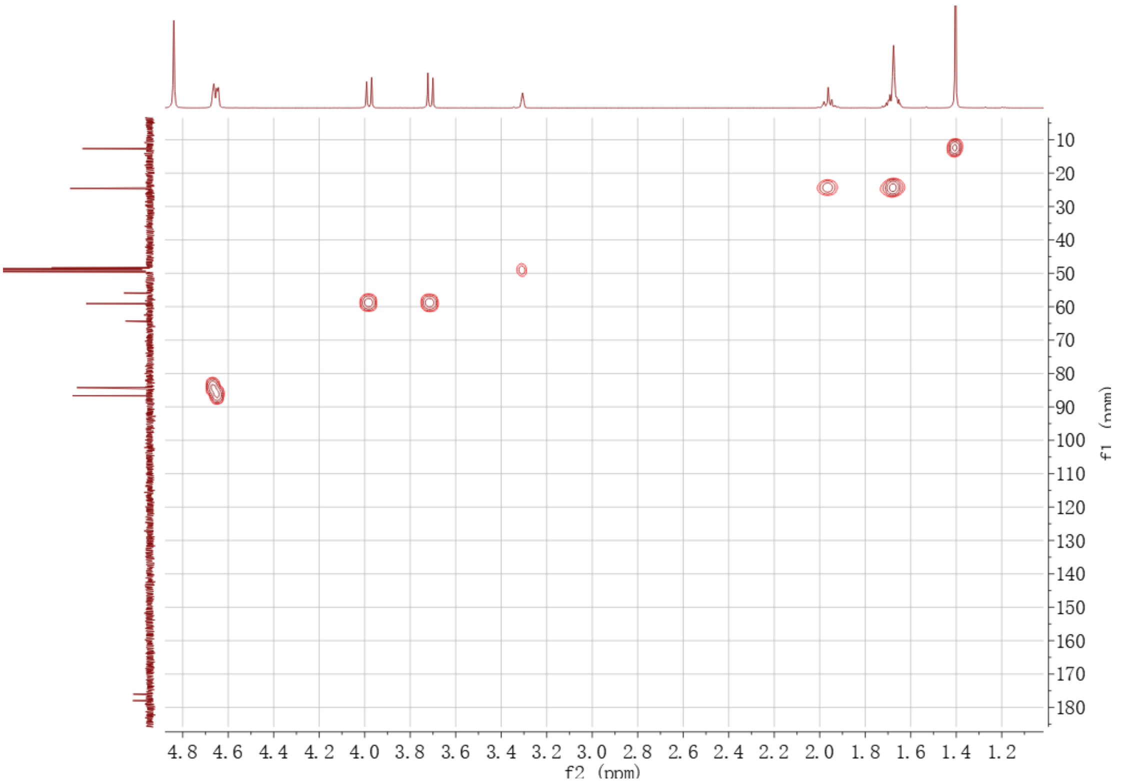

Figure S35. HSQC spectrum of **5** in CD_3OD

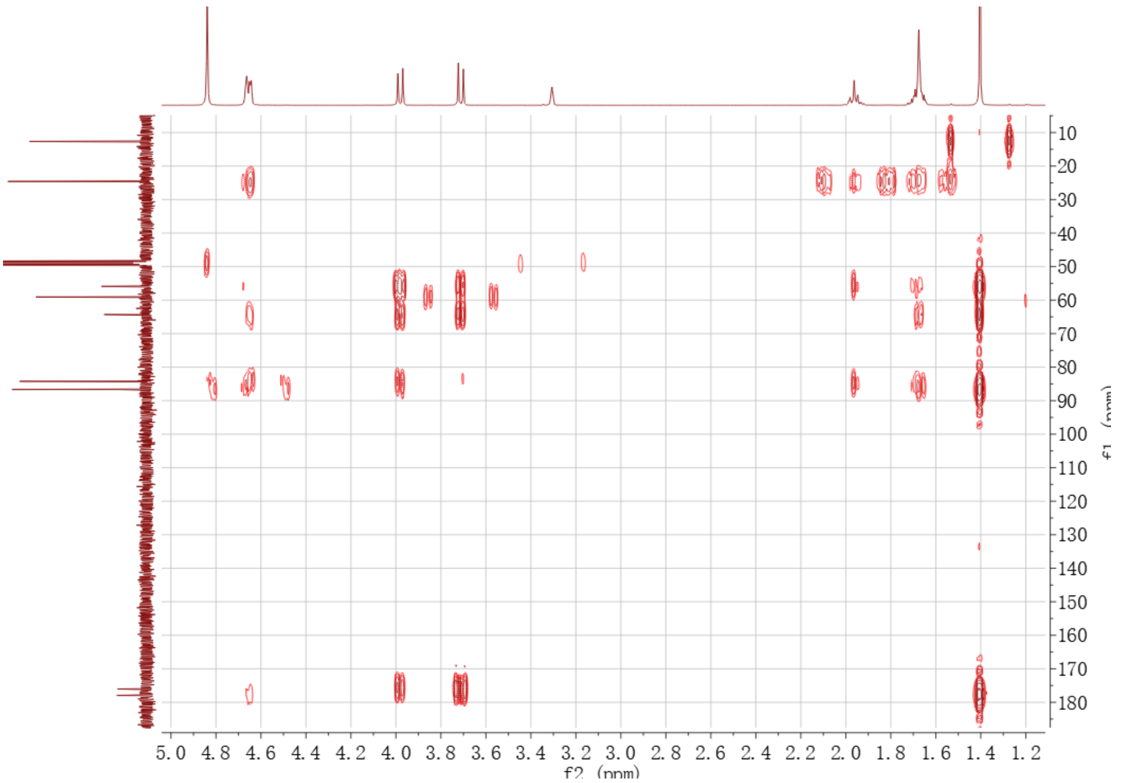

Figure S36. HMBC spectrum of **5** in CD_3OD

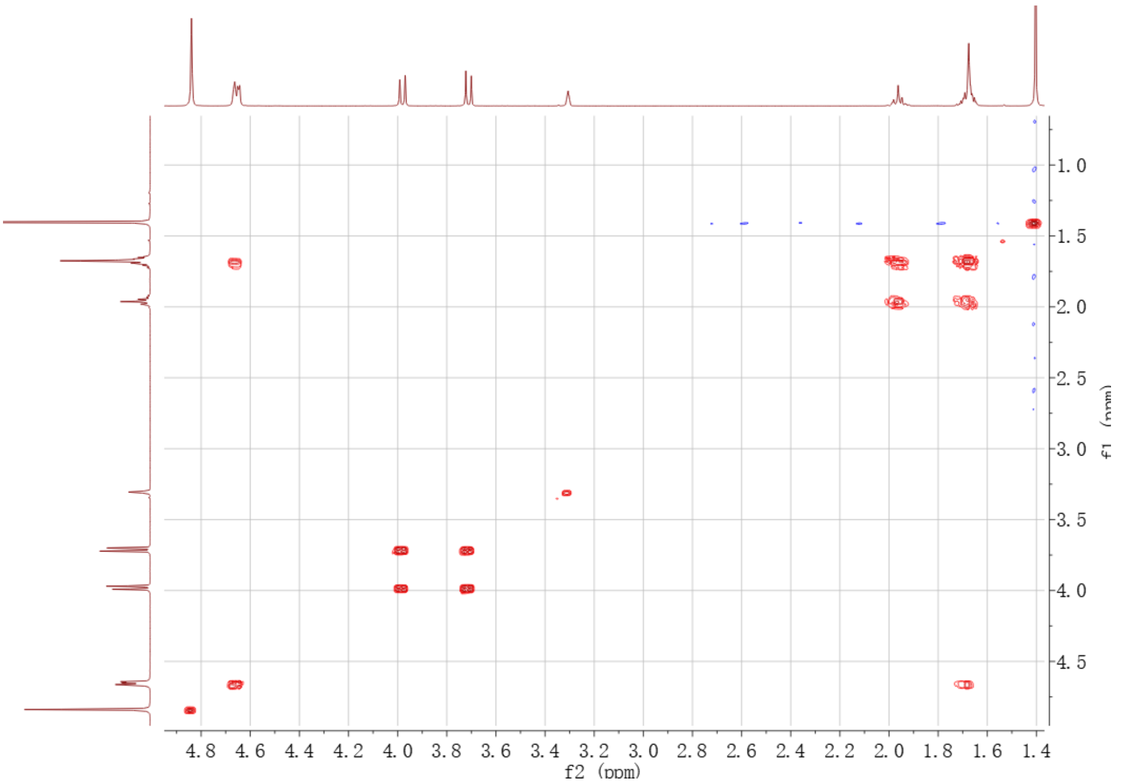

Figure S37. ^{1}H-^{1}H COSY spectrum of **5** in CD_3OD

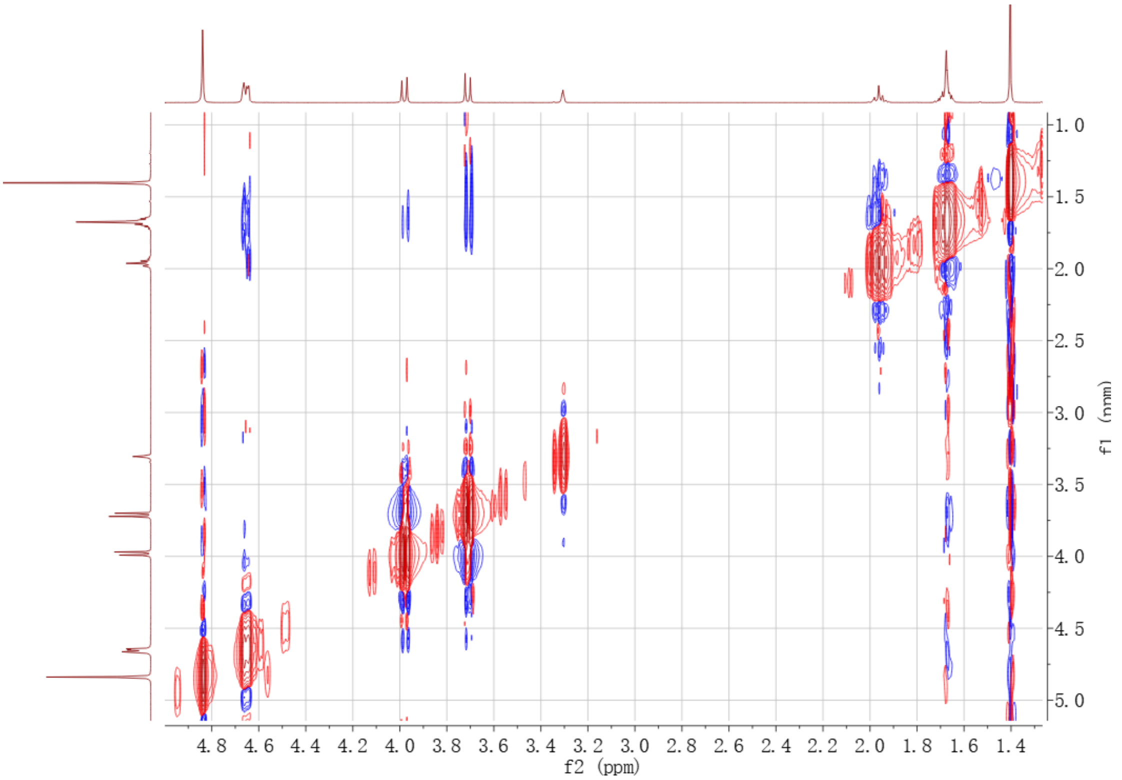

Figure S38. ROESY spectrum of **5** in CD_3OD

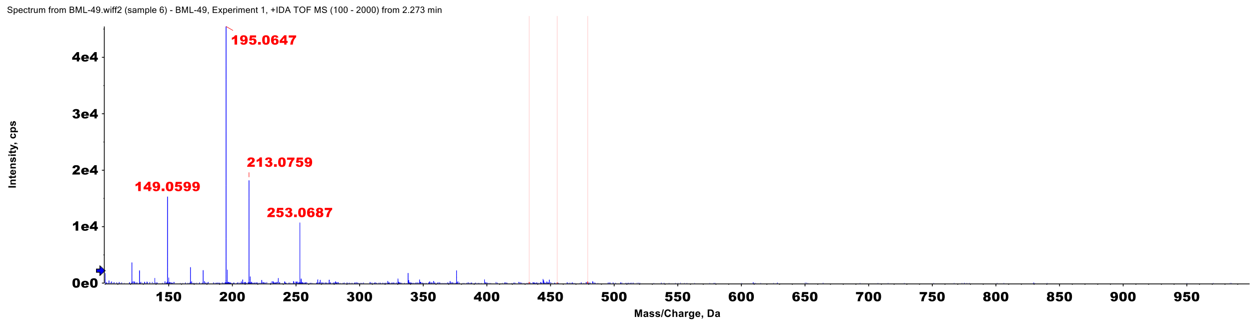

Hit	Formula	m/z	RDB	ppm	MS Rank	Found
1	C10H12O5	213.0757	5.0	0.7	1	NA/NA

Figure S39. HRESIMS of **5**

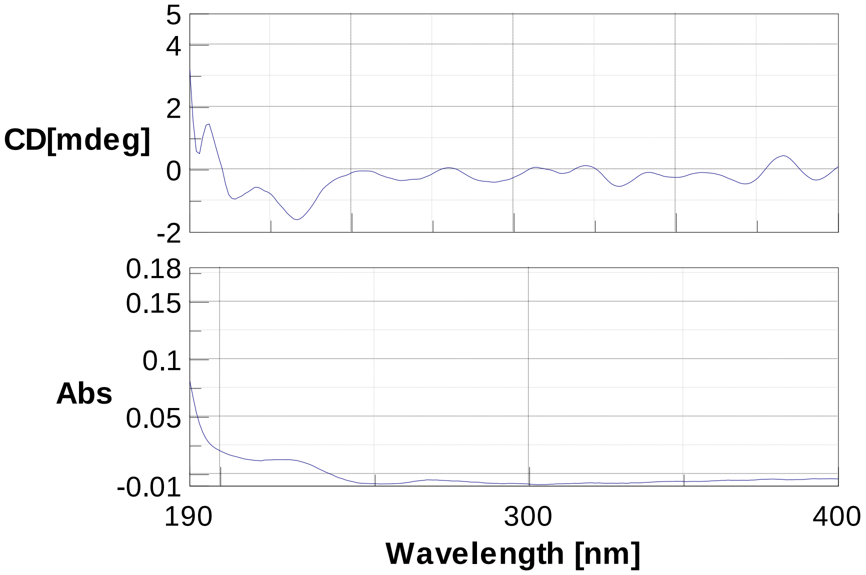

Figure S40. CD spectrum of **5**

Semi-synthesis of the compounds 2a and 2b

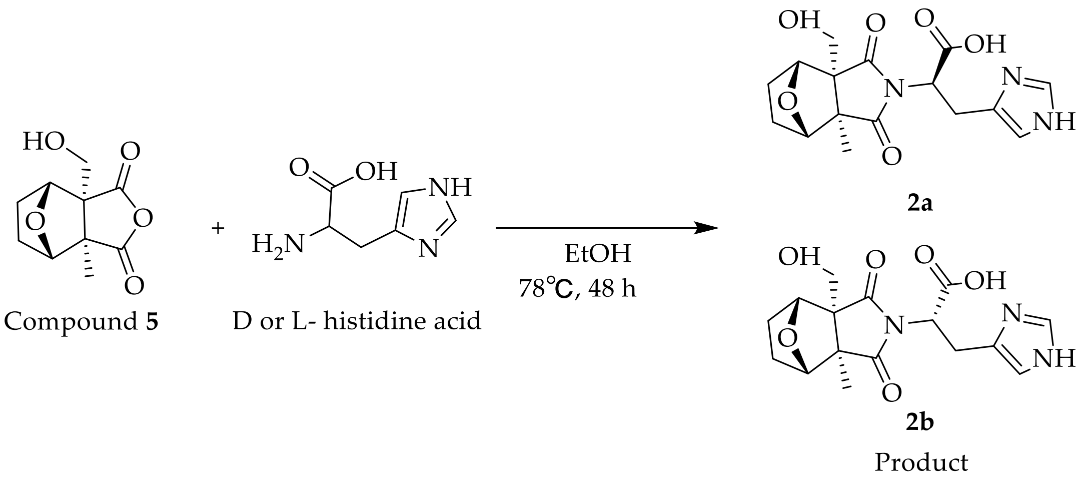

Scheme S1. Semi-synthesis of compounds **2a** and **2b**

2a: The D-histidine (10.0 mg, 0.065 mmol) and compound **5** (10.0 mg, 0.047 mmol) could react directly in the 95% EtOH of solvent at 78 ℃ for 48 h led to the generation of **2a** (Scheme 1). The yellow gum by vacuum filtration to afford **2a** (0.01 g, 90 %). ^{1}H NMR (500 MHz, CD_3OD): δ_H 8.46 (1H, s), 7.11 (1H, s), 4.80 (1H, dd, *J* = 10.5, 5.0 Hz), 4.52 (1H, d, *J* = 4.7 Hz, 2H), 4.29 (2H, d, *J* = 4.5 Hz), 3.86 (1H, d, *J* = 11.6 Hz), 3.70 (1H, d, *J* = 11.6 Hz), 3.48 (2H, m), 1.91 (1H, m), 1.76 (1H, m), 1.61 (2H, m), 1.23 (3H, s); ^{13}C NMR (125 MHz, CD_3OD): δ_C 182.9, 181.5, 169.0, 134.7, 132.1, 119.3, 85.2, 83.3, 61.0, 59.8, 55.3, 54.8, 25.8, 24.9, 24.8, 12.2; MS (ESI) *m/z*: 350.13 $[M+H]^+$

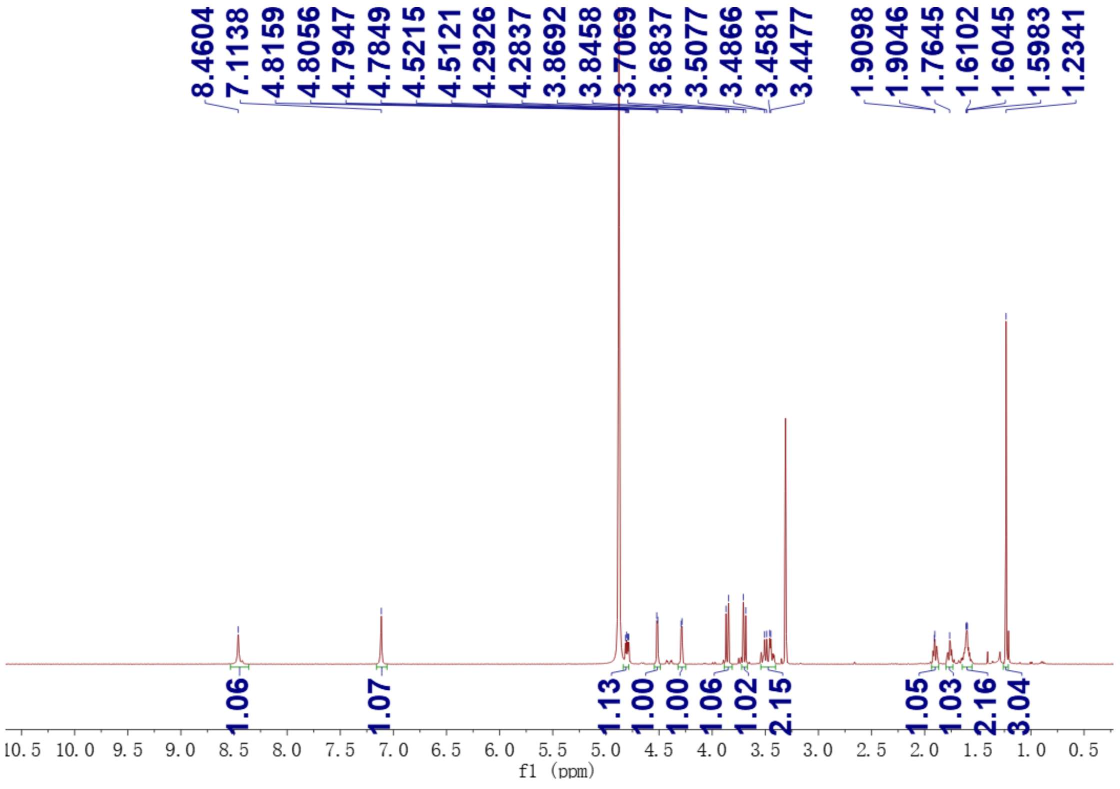

Figure S41. ^1H NMR spectrum of **2a** in CD_3OD

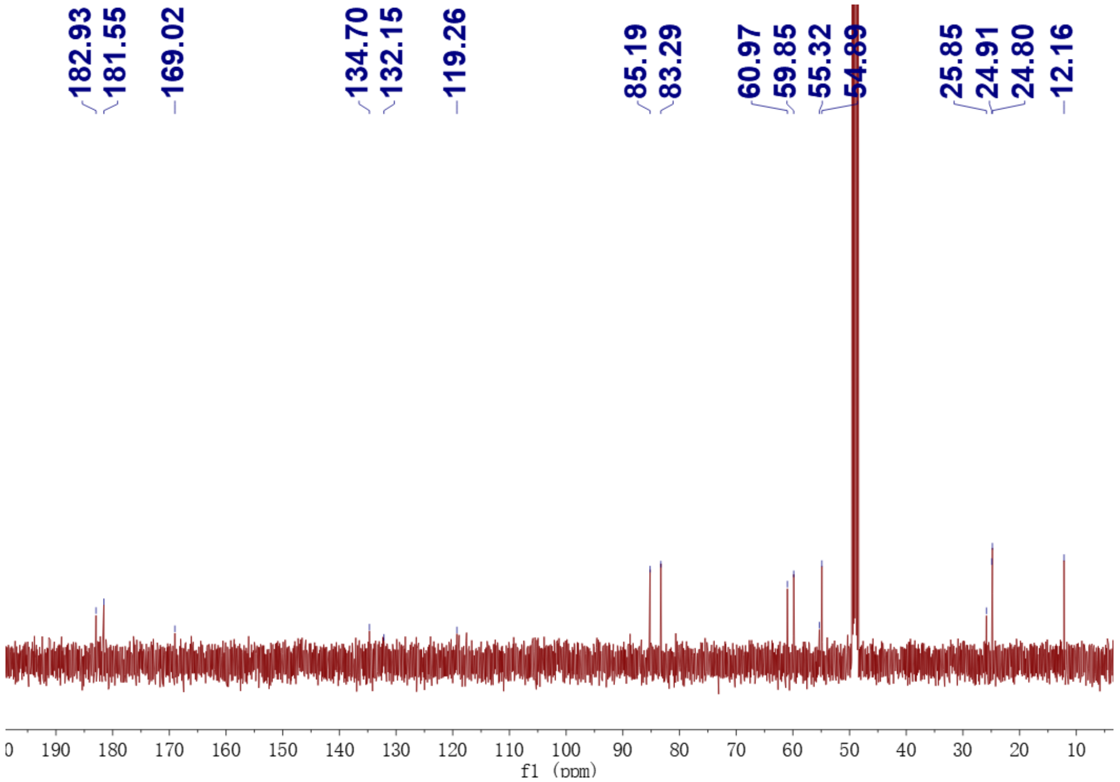

Figure S42. ^{13}C NMR spectrum of **2a** in CD_3OD

2b: The L-histidine (10.0 mg, 0.065 mmol) and compound **5** (10.0 mg, 0.047 mmol) could react directly in the 95% EtOH of solvent at 78 ℃ for 48 h led to the generation of **2b** (Scheme 1). The yellow gum by vacuum filtration to afford **2b** (0.01 g, 90 %). ^1H NMR (500 MHz, CD_3OD): δ_H 8.46 (1H, s), 7.11 (1H, s), 4.78 (1H, dd, *J* = 8.7, 6.9 Hz), 4.42 (1H, d, *J* = 4.6 Hz, 2H), 4.39 (2H, d, *J* = 4.6 Hz), 3.88 (1H, d, *J* = 11.5 Hz), 3.74 (1H, d, *J* = 11.5 Hz), 3.45 (2H, m), 1.89 (1H, m), 1.70 (1H, m), 1.60 (2H, m), 1.21 (3H, s); ^{13}C NMR (125 MHz, CD_3OD): δ_C 182.8, 181.2, 172.0, 134.7, 132.3, 119.1, 85.2, 83.4, 60.9, 59.9, 55.3, 54.5, 25.9, 25.2, 24.5, 12.0; MS (ESI) *m*/*z*: 350.13 $[M+H]^+$

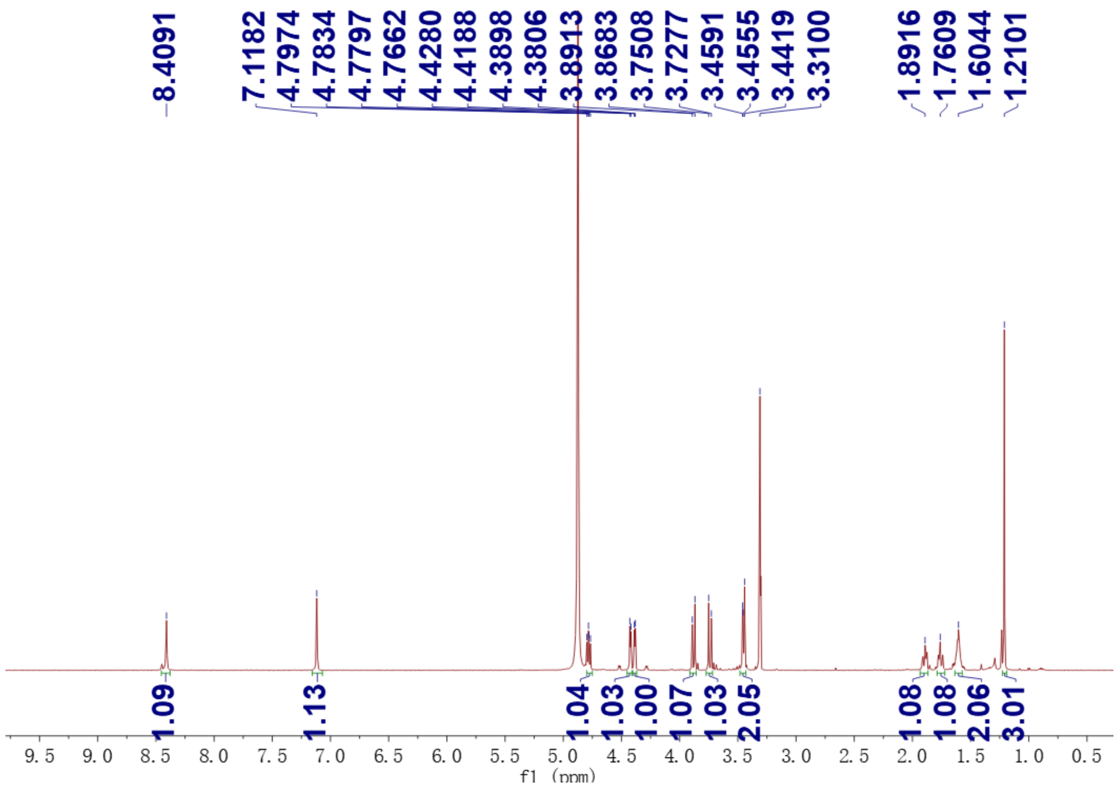

Figure S43. ^{1}H NMR spectrum of **2b** in CD_3OD

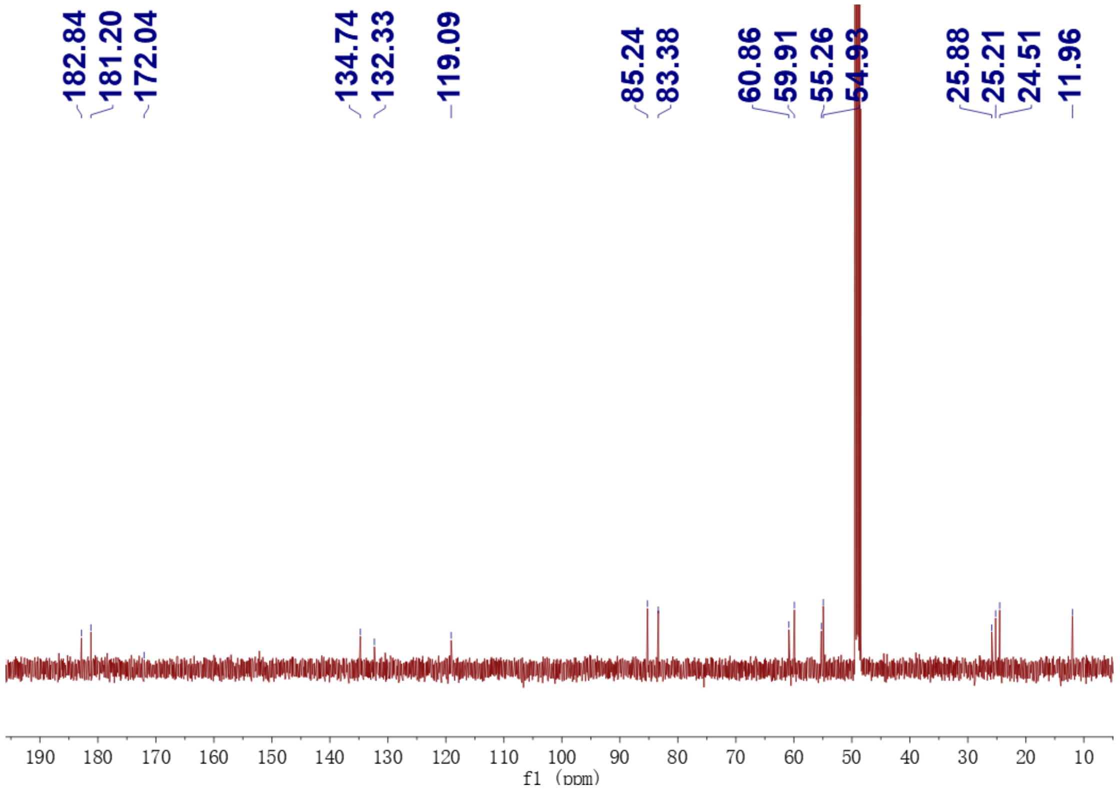

Figure S44. ^{13}C NMR spectrum of **2b** in CD_3OD

Figure S45. HPLC analyses of compounds **2**, **2a** and 2**b**

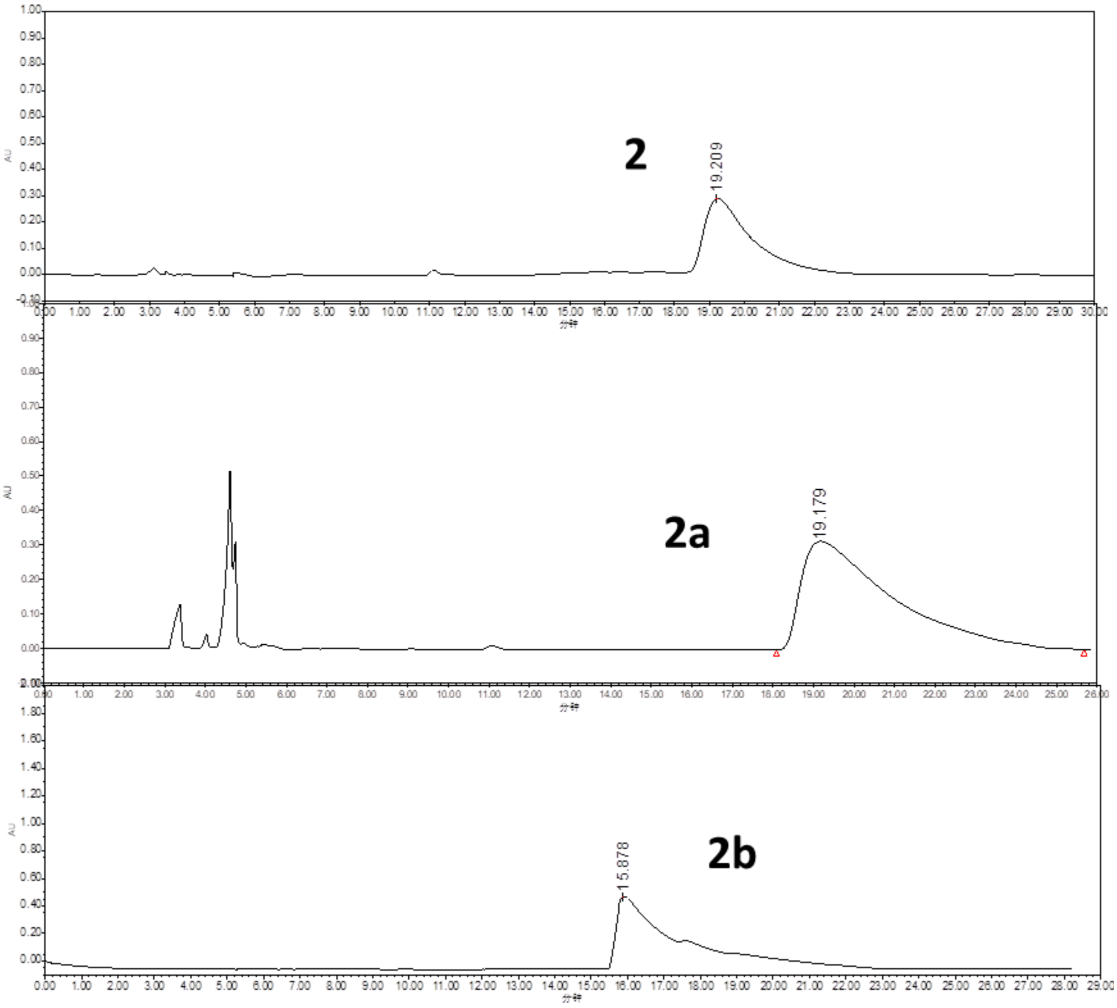

[The analysis was performed on Waters 2535 spectrometer using Waters Atlantis™ T3 OBD™ Prep column (250 mm × 10 mm, 5 μm). MeCN/H_2O, 2%, flow rate: 3 mL/min]

ECD calculation for compounds **1–5**

Conformation search using molecular mechanics calculations was performed in CONFLEX version 7.0 with MMFF force field with an energy window for acceptable conformers (ewindow) of 5 kcal/mol above the ground state, a maximum number of conformations per molecule (maxconfs) of 100, and an RMSD cutoff (rmsd) of 0.5Å. Then the predominant conformers were optimized at B3LYP/6-31 (d,p) level in Gaussian 09. The optimized conformation geometries and thermodynamic parameters of all selected conformations were provided. The optimized conformers of **1–5** were used for the ECD calculation, which were performed with Gaussian 09 B3LYP/6-31 (d,p). The solvent effects were taken into account by the polarizable-conductor calculation model (PCM, methanol as the solvent).

Figure S46. Optimized geometries of predominant conformers for (1*R*,2*R*,3*S*,6*R*)-**1** at the B3LYP/6-31 (d,p) level

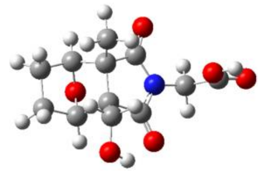

Conf 1

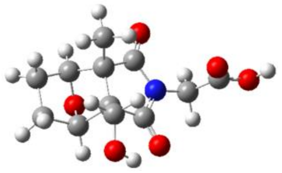

Conf 2

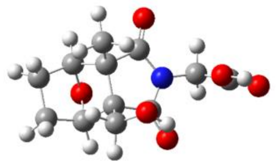

Conf 3

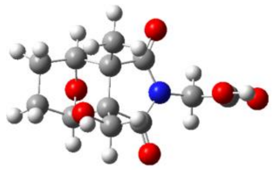

Conf 4

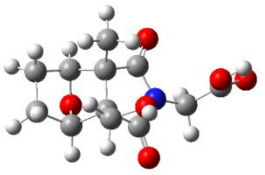

Conf 5

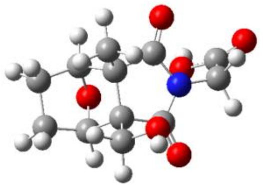

Conf 6

Figure S47. Optimized geometries of predominant conformers for (1*R*,2*R*,3*S*,6*R*,1′*R*)-**2** at the B3LYP/6-31 (d,p) level

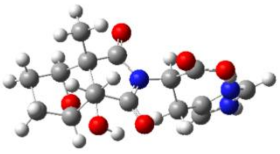

Conf 1

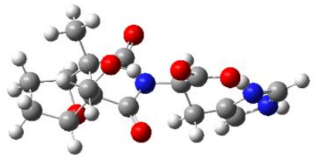

Conf 2

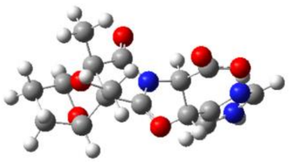

Conf 3

Figure S48 Optimized geometries of predominant conformers for (1*S*,2*R*,3*S*,6*R*)-**3** at the B3LYP/6-31 (d,p) level

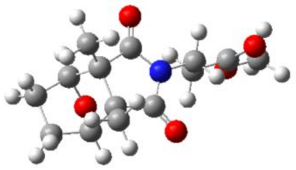

Conf 1

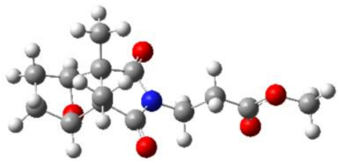

Conf 2

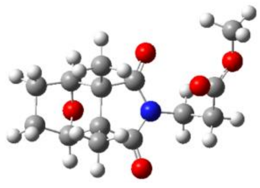

Conf 3

Figure S49. Optimized geometries of predominant conformers for (1*S*,2*R*,3*S*,6*R*)-**4** at the B3LYP/6-31 (d,p) level

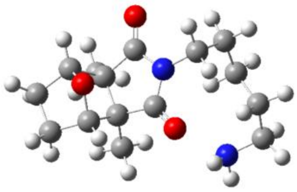

Conf 1

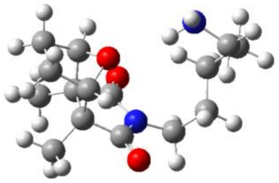

Conf 2

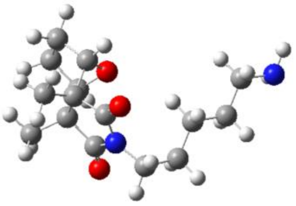

Conf 3

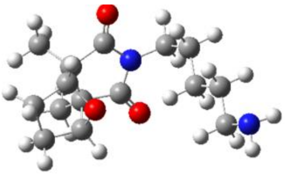

Conf 4

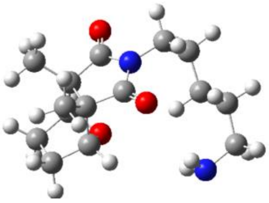

Conf 5

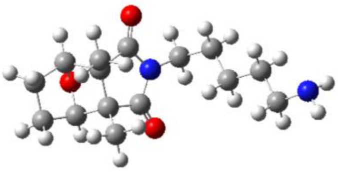

Conf 6

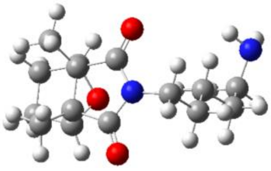

Conf 7

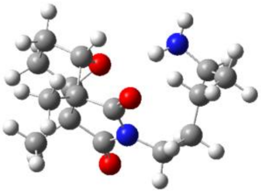

Conf 8

Figure S50. Optimized geometries of predominant conformers for (1*R*,2*R*,3*S*,6*R*)-**5** at the B3LYP/6-31 (d,p) level

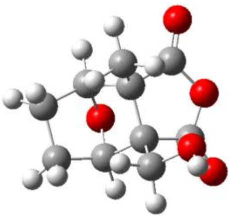

Conf 1

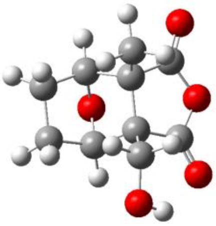

Conf 2

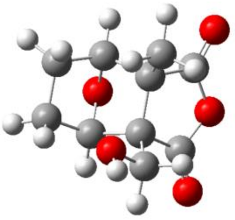

Conf 3

Table S1. The Cartesian coordinates of the lowest energy conformers for (1*R*,2*R*,3*S*,6*R*)-**1**

**Conf 1**	**X axis(Å)**	**Y axis(Å)**	**Z axis(Å)**	**Conf 2**	**X axis(Å)**	**Y axis(Å)**	**Z axis(Å)**
C	3.4551	0.2815	-0.3494	C	3.4661	0.2633	-0.3555
C	3.3577	-1.1853	0.0815	C	3.3607	-1.2022	0.0779
C	1.9271	-1.5481	-0.3187	C	1.9267	-1.5565	-0.3178
C	0.8656	-0.8807	0.5731	C	0.8719	-0.8814	0.576
C	0.9718	0.5988	0.1387	C	0.9864	0.5971	0.1397
C	2.0671	0.54	-0.9377	C	2.0781	0.5298	-0.9399
C	-0.5042	-1.3086	0.0865	C	-0.5018	-1.301	0.0937
N	-1.1	-0.2662	-0.5557	N	-1.0959	-0.2532	-0.5415
C	-0.3564	0.8726	-0.5308	C	-0.3419	0.8791	-0.5261
O	-0.7019	1.9709	-0.9484	O	-0.6789	1.9764	-0.9531
O	-0.9932	-2.4148	0.2674	O	-0.9954	-2.406	0.2692
O	1.8084	-0.7611	-1.5347	O	1.8094	-0.7705	-1.5347
C	1.1685	1.6513	1.223	C	1.1935	1.6498	1.222
C	0.9782	-1.1958	2.0594	C	0.9861	-1.1955	2.0623
C	-2.4448	-0.3286	-1.0928	C	-2.4487	-0.302	-1.0579
C	-3.4867	0.0407	-0.0503	C	-3.4004	0.0766	0.0612
O	1.5041	2.9136	0.6552	O	1.5392	2.9077	0.6497
O	-2.9955	0.3705	1.1639	O	-4.694	-0.015	-0.3118
O	-4.6942	0.0613	-0.2384	O	-3.1071	0.4434	1.1892
H	4.2202	0.3759	-1.129	H	3.7448	0.9356	0.4591
H	3.7269	0.9544	0.4669	H	4.2295	0.3516	-1.1375
H	4.074	-1.7767	-0.5011	H	4.0716	-1.7991	-0.5058
H	3.5815	-1.3423	1.1388	H	3.5864	-1.3591	1.1348
H	1.7688	-2.6107	-0.5209	H	1.7613	-2.6184	-0.518
H	2.0318	1.3303	-1.6921	H	2.0456	1.3193	-1.6954
H	0.2683	1.7825	1.8345	H	0.2957	1.7902	1.8349
H	1.9848	1.387	1.9006	H	2.009	1.3802	1.8983
H	0.1393	-0.765	2.6183	H	0.1518	-0.7581	2.6229
H	0.9563	-2.2782	2.2306	H	0.9569	-2.2775	2.2348
H	1.9014	-0.8128	2.5024	H	1.9132	-0.8186	2.5025
H	-2.65	-1.3488	-1.4308	H	-2.6776	-1.3184	-1.391
H	-2.5319	0.3781	-1.9236	H	-2.5591	0.4163	-1.8754
H	0.7431	3.2019	0.1085	H	0.7721	3.2077	0.1181
H	-3.7997	0.5703	1.69	H	-5.1968	0.253	0.4867
**Conf 3**	**X axis(Å)**	**Y axis(Å)**	**Z axis(Å)**	**Conf 4**	**X axis(Å)**	**Y axis(Å)**	**Z axis(Å)**
C	3.5037	0.4903	-0.564	C	3.3871	0.2281	-0.7516
C	3.5169	-0.817	0.2339	C	3.3235	-1.0667	0.0626
C	2.1153	-1.3695	-0.0293	C	1.8743	-1.5113	-0.1447
C	1.0131	-0.5654	0.6871	C	0.8608	-0.6517	0.6294
C	0.9891	0.7406	-0.1407	C	0.9208	0.6873	-0.1456
C	2.1004	0.4866	-1.1729	C	1.9503	0.3693	-1.2475

C	-0.3242	-1.2097	0.3873	C	-0.5292	-1.1805	0.3284
N	-0.9922	-0.4585	-0.5322	N	-1.1717	-0.3192	-0.5076
C	-0.3376	0.6873	-0.8655	C	-0.4403	0.7898	-0.7996
O	-0.7575	1.5658	-1.607	O	-0.8179	1.7412	-1.4707
O	-0.7282	-2.2471	0.8941	O	-0.9969	-2.2165	0.7812
O	1.9435	-0.9391	-1.4069	O	1.6622	-1.0359	-1.5039
C	1.0843	2.0795	0.5799	C	1.1378	1.991	0.6161
C	1.1934	-0.4682	2.1972	C	1.0411	-0.6418	2.1422
C	-2.3195	-0.7859	-1.013	C	-2.5375	-0.5136	-0.9513
C	-3.4058	-0.1783	-0.1415	C	-3.5387	0.09	0.0189
O	0.0396	2.238	1.5338	O	2.2776	1.9512	1.4589
O	-2.9662	0.6088	0.8637	O	-3.0018	0.7066	1.0938
O	-4.6072	-0.3381	-0.3015	O	-4.7536	0.0573	-0.1125
H	3.7301	1.3699	0.0417	H	3.767	1.0841	-0.1932
H	4.2588	0.4314	-1.357	H	4.0605	0.0746	-1.6041
H	4.2731	-1.4877	-0.1911	H	4.0111	-1.7995	-0.3762
H	3.7541	-0.6779	1.2909	H	3.6064	-0.9346	1.1088
H	2.0335	-2.4563	0.0564	H	1.7186	-2.5914	-0.0788
H	2.0115	1.0493	-2.1061	H	1.8484	0.957	-2.164
H	2.0226	2.1698	1.1333	H	1.2652	2.8263	-0.0825
H	1.0416	2.9225	-0.1194	H	0.2699	2.2204	1.2457
H	0.2938	-0.0776	2.6855	H	0.2699	-0.0316	2.6264
H	1.3791	-1.4587	2.6287	H	0.9507	-1.6549	2.5516
H	2.0314	0.1768	2.4766	H	2.0147	-0.2606	2.4566
H	-2.4472	-1.8725	-1.0055	H	-2.7425	-1.5858	-1.0261
H	-2.4403	-0.3913	-2.0264	H	-2.6721	-0.0274	-1.9222
H	-0.7639	2.5212	1.0562	H	2.3512	2.8235	1.8875
H	-3.7893	0.885	1.3209	H	-3.7858	1.0168	1.5956
**Conf 5**	**X axis(Å)**	**Y axis(Å)**	**Z axis(Å)**	**Conf 6**	**X axis(Å)**	**Y axis(Å)**	**Z axis(Å)**
C	3.5101	0.4203	-0.5915	C	2.9067	-1.1334	-1.2282
C	3.4968	-0.869	0.2352	C	2.7974	-1.8768	0.1075
C	2.0813	-1.3939	-0.0101	C	1.3344	-1.6409	0.4869
C	1.0023	-0.5499	0.697	C	1.0557	-0.18	0.8933
C	1.0035	0.7393	-0.1565	C	1.1236	0.5427	-0.4715
C	2.1025	0.438	-1.1909	C	1.4834	-0.6067	-1.4265
C	-0.3506	-1.1702	0.4171	C	-0.3961	-0.0467	1.3025
N	-1.0098	-0.4214	-0.5103	N	-1.084	0.613	0.3295
C	-0.3275	0.6985	-0.8802	C	-0.312	0.9441	-0.7433
O	-0.7058	1.5376	-1.6879	O	-0.7017	1.4776	-1.7742
O	-0.7643	-2.1988	0.9353	O	-0.8658	-0.4799	2.3463
O	1.9137	-0.9885	-1.3952	O	0.7399	-1.7052	-0.8357
C	1.1329	2.0928	0.5295	C	1.9896	1.7873	-0.6115
C	1.1981	-0.4321	2.2037	C	1.9383	0.3222	2.0311

C	-2.3323	-0.7494	-1.0031	C	-2.51	0.864	0.3925
C	-3.436	-0.1628	-0.1407	C	-3.3317	-0.2751	-0.1849
O	0.1298	2.2491	1.5254	O	1.6194	2.7627	0.3574
O	-3.0179	0.5364	0.9327	O	-2.6188	-1.3224	-0.6414
O	-4.6338	-0.2878	-0.3534	O	-4.5527	-0.294	-0.2523
H	3.7642	1.3067	-0.0072	H	3.6776	-0.3605	-1.231
H	4.2573	0.3247	-1.3884	H	3.1563	-1.8528	-2.0172
H	4.2353	-1.5663	-0.178	H	2.9752	-2.9454	-0.0631
H	3.7421	-0.712	1.2878	H	3.5181	-1.5365	0.8541
H	1.9753	-2.4766	0.0987	H	0.9096	-2.401	1.1478
H	2.0216	0.9834	-2.135	H	1.1992	-0.4567	-2.4715
H	2.0972	2.2074	1.0308	H	3.0494	1.5693	-0.4572
H	1.0301	2.9116	-0.1916	H	1.8792	2.2349	-1.6057
H	0.3054	-0.0314	2.6965	H	1.5668	1.2662	2.4449
H	1.3834	-1.4174	2.6474	H	1.9486	-0.3976	2.858
H	2.0422	0.2129	2.4645	H	2.9738	0.4806	1.7165
H	-2.4522	-1.8372	-1.0077	H	-2.7386	1.7673	-0.1815
H	-2.4473	-0.3487	-2.0149	H	-2.8037	0.9927	1.4388
H	0.1298	3.1847	1.7973	H	2.0897	3.587	0.1347
H	-3.8439	0.8299	1.3698	H	-3.2865	-1.9571	-0.9735

Table S2. The Cartesian coordinates of the lowest energy conformers for (1*R*,2*R*,3*S*,6*R*,1′*R*)-**2**

Conf 1	X axis(Å)	Yaxis(Å)	Z axis(Å)	Conf 2	Xaxis(Å)	Yaxis(Å)	Zaxis(Å)
C	-4.2598	-1.3684	0.6065	C	4.3559	-1.2947	-0.8225
C	-4.2515	-1.5035	-0.9192	C	4.3142	-1.622	0.6736
C	-2.7643	-1.3702	-1.2491	C	2.8186	-1.5306	0.9775
C	-2.2296	0.0583	-1.046	C	2.2868	-0.085	0.8946
C	-2.2403	0.1856	0.4914	C	2.2455	0.1443	-0.6319
C	-2.7747	-1.1885	0.9248	C	2.8725	-1.148	-1.1701
C	-0.7586	0.0722	-1.4039	C	0.8292	-0.0475	1.2972
N	-0.0098	0.15	-0.2663	N	0.0466	0.0374	0.1828
C	-0.7756	0.272	0.8551	C	0.7744	0.0961	-0.9698
O	-0.3671	0.4401	1.9977	O	0.3237	0.1383	-2.1066
O	-0.3251	0.0268	-2.5469	O	0.4264	-0.1002	2.4512
O	-2.1981	-2.051	-0.0959	O	2.302	-2.1192	-0.2468
C	-2.9423	1.3903	1.1066	C	2.7954	1.4453	-1.2037
C	2.17	-0.3839	0.8561	C	-2.1861	-0.5446	-0.8038
C	3.5708	-0.7692	0.5112	C	-3.5926	-0.8139	-0.3801
C	1.4489	0.3204	-0.2997	C	-1.4019	0.2576	0.2399
C	1.7038	1.8373	-0.3085	C	-1.593	1.7762	0.0681
C	-2.9408	1.1359	-1.8548	C	3.0657	0.901	1.7611
C	4.2973	-1.8631	0.9263	C	-4.3932	-1.8967	-0.6688
N	5.5192	-1.7369	0.3259	N	-5.5832	-1.6457	-0.0441

C	5.4944	-0.5915	-0.4181	C	-5.4681	-0.4409	0.5894
N	4.3327	0.0185	-0.3266	N	-4.2789	0.09	0.4048
O	-3.0948	1.2239	2.5136	O	2.2755	2.5855	-0.5275
O	2.9908	2.2025	-0.5058	O	-2.8459	2.2248	0.3074
O	0.8214	2.6811	-0.18	O	-0.6917	2.5504	-0.2473
H	-4.8949	-0.5549	0.9644	H	4.9562	-0.4123	-1.0533
H	-4.6314	-2.3011	1.047	H	4.7906	-2.1438	-1.3629
H	-4.6099	-2.5035	-1.191	H	4.6662	-2.6498	0.824
H	-4.8902	-0.7748	-1.4227	H	4.9416	-0.9653	1.2801
H	-2.4616	-1.8565	-2.1802	H	2.4975	-2.0994	1.854
H	-2.4802	-1.5169	1.9251	H	2.6236	-1.3985	-2.2048
H	-2.3918	2.3216	0.9306	H	3.882	1.4954	-1.0964
H	-3.9476	1.525	0.6984	H	2.5754	1.5515	-2.2719
H	1.6285	-1.2989	1.1294	H	-1.6935	-1.5103	-0.976
H	2.2022	0.2557	1.7465	H	-2.217	-0.0169	-1.7647
H	1.792	-0.0623	-1.2694	H	-1.7261	0.0085	1.2581
H	-2.4493	2.1083	-1.7341	H	2.5282	1.8476	1.8844
H	-2.9213	0.8966	-2.9243	H	3.2134	0.4955	2.769
H	-3.9881	1.2573	-1.5656	H	4.0519	1.1291	1.346
H	4.0592	-2.6931	1.5775	H	-4.2251	-2.7949	-1.2474
H	6.3025	-2.3707	0.4146	H	-6.4028	-2.2384	-0.0487
H	6.3466	-0.2586	-0.9973	H	-6.2788	-0.0081	1.162
H	-2.1957	1.2346	2.904	H	1.3276	2.7075	-0.7555
H	3.6203	1.438	-0.5717	H	-3.499	1.5014	0.496

**Conf 3**	**X axis(Å)**	**Y axis(Å)**	**Z axis(Å)**
C	4.1433	-1.5947	-0.6392
C	4.1648	-1.5536	0.8907
C	2.6894	-1.3356	1.2305
C	2.2011	0.0887	0.9153
C	2.1807	0.0844	-0.6297
C	2.6705	-1.3401	-0.9506
C	0.7363	0.1809	1.2937
N	-0.0341	0.1713	0.1683
C	0.7092	0.1496	-0.9744
O	0.2727	0.1759	-2.1178
O	0.3192	0.2505	2.4422
O	2.0741	-2.091	0.1485
C	2.8548	1.2206	-1.3918
C	-2.2463	-0.4524	-0.8377
C	-3.6429	-0.7725	-0.4167
C	-1.4881	0.3723	0.2097
C	-1.7205	1.8837	0.0422
C	2.9445	1.2023	1.6433

C	-4.3885	-1.9024	-0.6701
N	-5.5951	-1.6848	-0.0649
C	-5.5432	-0.4528	0.5227
N	-4.3785	0.1265	0.3275
O	4.2146	1.3942	-1.0268
O	-2.9977	2.2911	0.2227
O	-0.8311	2.6922	-0.2071
H	4.8395	-0.9002	-1.11
H	4.423	-2.6034	-0.9677
H	4.5009	-2.526	1.2699
H	4.8358	-0.7929	1.2945
H	2.3866	-1.7295	2.2043
H	2.341	-1.7409	-1.9131
H	2.8202	1.0334	-2.4714
H	2.3373	2.1697	-1.2075
H	-1.7237	-1.4017	-1.0131
H	-2.2928	0.0779	-1.7966
H	-1.817	0.1149	1.2246
H	2.5189	2.1832	1.4021
H	2.8647	1.0778	2.7297
H	4.0099	1.2338	1.4062
H	-4.1723	-2.813	-1.2121
H	-6.3855	-2.3158	-0.0528
H	-6.3788	-0.0374	1.0719
H	4.5488	2.1611	-1.5274
H	-3.6354	1.5497	0.3918

Table S3. The Cartesian coordinates of the lowest energy conformers for (1*S*,2*R*,3*S*,6*R*)-**3**

**Conf 1**	**X axis(Å)**	**Y axis(Å)**	**Z axis(Å)**	**Conf 2**	**X axis(Å)**	**Y axis(Å)**	**Z axis(Å)**
C	-1.4051	-3.7589	-0.7659	C	0.2557	-4.2799	0.7661
C	-1.4051	-3.7589	0.7659	C	0.2557	-4.2799	-0.7661
C	-1.0463	-2.3081	1.0905	C	0.6411	-2.8357	-1.0905
C	0.4202	-1.9675	0.7724	C	-0.4797	-1.8303	-0.7725
C	0.4202	-1.9675	-0.7724	C	-0.4797	-1.8303	0.7725
C	-1.0463	-2.3081	-1.0905	C	0.6411	-2.8357	1.0905
C	0.6649	-0.5182	1.1389	C	0.0029	-0.4409	-1.1381
N	0.8069	0.2198	0	N	0.2925	0.2536	0
C	0.6649	-0.5182	-1.1389	C	0.0029	-0.4409	1.1381
O	0.7173	-0.0929	-2.2846	O	0.0871	-0.0152	2.2819
O	0.7173	-0.0929	2.2846	O	0.0871	-0.0152	-2.2819
O	-1.7358	-1.6385	0	O	1.5677	-2.5838	0
C	1.4536	-2.833	-1.4827	C	-1.8026	-2.0934	1.4819
C	1.4536	-2.833	1.4827	C	-1.8026	-2.0934	-1.4819

C	-0.0456	3.9012	0	C	0.1987	4.0338	0
C	-0.2926	2.4173	0	C	-0.3412	2.6295	0
C	1.0274	1.6562	0	C	0.7969	1.6169	0
O	-1.2372	4.5626	0	O	-0.8378	4.9193	0
O	1.0548	4.4343	0	O	1.3829	4.337	0
C	-1.1135	5.9855	0	C	-0.432	6.2889	0
H	-0.7269	-4.4968	-1.1997	H	-0.6941	-4.5996	1.1999
H	-2.4165	-3.9867	-1.123	H	1.032	-4.9671	1.1231
H	-2.4165	-3.9867	1.123	H	1.032	-4.9671	-1.1231
H	-0.7269	-4.4968	1.1997	H	-0.6941	-4.5996	-1.1999
H	-1.4103	-1.9579	2.06	H	1.1285	-2.7035	-2.06
H	-1.4103	-1.9579	-2.06	H	1.1285	-2.7035	2.06
H	1.2982	-2.8129	-2.5675	H	-1.6572	-2.1534	2.5666
H	2.4701	-2.4669	-1.2974	H	-2.5173	-1.2829	1.298
H	1.4166	-3.8783	-1.1642	H	-2.2734	-3.0268	1.1613
H	2.4701	-2.4669	1.2974	H	-2.5173	-1.2829	-1.298
H	1.2982	-2.8129	2.5675	H	-1.6572	-2.1534	-2.5666
H	1.4166	-3.8783	1.1642	H	-2.2734	-3.0268	-1.1613
H	-0.8766	2.1589	0.8904	H	-0.9655	2.4944	0.8903
H	-0.8766	2.1589	-0.8904	H	-0.9655	2.4944	-0.8903
H	1.6161	1.9205	-0.8865	H	1.4269	1.7564	-0.8865
H	1.6161	1.9205	0.8865	H	1.4269	1.7564	0.8865
H	-2.1199	6.4131	0	H	0.1459	6.5174	0.901
H	-0.5928	6.3249	0.9009	H	0.1459	6.5174	-0.901
H	-0.5928	6.3249	-0.9009	H	-1.3328	6.909	0

**Conf 3**	**X axis(Å)**	**Y axis(Å)**	**Z axis(Å)**
C	4.023	-0.0497	0.2961
C	3.614	-1.4757	-0.0864
C	2.2675	-1.2529	-0.7769
C	1.1506	-0.8541	0.2028
C	1.5631	0.5837	0.5891
C	2.8495	0.7771	-0.2319
C	-0.1328	-0.6694	-0.58
N	-0.4338	0.6606	-0.6521
C	0.4744	1.4504	-0.0093
O	0.4221	2.669	0.0893
O	-0.7915	-1.5826	-1.0596
O	2.5395	0.0164	-1.431
C	1.687	0.9111	2.0722
C	0.8949	-1.8465	1.3307
C	-3.1982	0.2712	0.3909
C	-2.7295	1.5137	-0.3128
C	-1.6241	1.1792	-1.3045

O	-4.1454	-0.3666	-0.347
O	-2.7493	-0.1123	1.4637
C	-4.5725	-1.6097	0.2113
H	4.2155	0.0748	1.3638
H	4.9422	0.2141	-0.2402
H	4.3428	-1.8767	-0.8008
H	3.5745	-2.1588	0.7647
H	1.9999	-2.022	-1.5063
H	3.0991	1.813	-0.4764
H	2.0651	1.9299	2.2154
H	0.7125	0.8574	2.5714
H	2.3642	0.2322	2.5976
H	0.0195	-1.555	1.9226
H	0.6926	-2.8466	0.9304
H	1.7426	-1.9317	2.0159
H	-3.5804	1.9804	-0.8218
H	-2.3901	2.2276	0.4462
H	-1.9784	0.4467	-2.0385
H	-1.343	2.0918	-1.8424
H	-5.3075	-2.0512	-0.4673
H	-3.7279	-2.2999	0.3032
H	-5.05	-1.4485	1.1827

Table S4. The Cartesian coordinates of the lowest energy conformers for (1*S*,2*R*,3*S*,6*R*)-**4**

Conf 1	X axis(Å)	Y axis(Å)	Z axis(Å)	Conf 2	X axis(Å)	Y axis(Å)	Z axis(Å)
C	3.9593	-0.3545	0.331	C	-2.3467	2.1465	-0.8921
C	3.4182	-1.6615	-0.2573	C	-2.4445	2.1763	0.6361
C	2.1202	-1.2041	-0.9249	C	-1.337	1.207	1.0524
C	1.0225	-0.8343	0.0876	C	-1.681	-0.2634	0.7543
C	1.5686	0.4801	0.685	C	-1.6135	-0.3017	-0.7877
C	2.889	0.6558	-0.0851	C	-1.2121	1.1483	-1.1236
C	-0.2131	-0.4061	-0.6782	C	-0.5051	-1.1111	1.193
N	-0.3851	0.9426	-0.5415	N	0.1667	-1.5583	0.0918
C	0.5892	1.5332	0.2108	C	-0.4292	-1.1983	-1.0818
O	0.6731	2.7239	0.4808	O	-0.0758	-1.5269	-2.2058
O	-0.9297	-1.1748	-1.3076	O	-0.2152	-1.3377	2.3599
O	2.5367	0.1099	-1.3852	O	-0.3622	1.4852	0.0096
C	1.6879	0.5739	2.2015	C	-2.8307	-0.8256	-1.5394
C	0.6357	-1.9455	1.0562	C	-2.9456	-0.7815	1.4257
C	-4.0053	-0.0766	-0.3081	C	4.0551	0.5204	-0.4206
C	-3.1946	0.8413	0.6122	C	2.9481	-0.4906	-0.7377
C	-2.5797	2.0395	-0.1189	C	2.6025	-1.3953	0.4507
C	-1.4845	1.6826	-1.1347	C	1.405	-2.3123	0.1698

C	-4.3725	-1.4091	0.3478	C	3.6596	1.6361	0.5526
N	-3.1826	-2.1941	0.6637	N	2.6128	2.4976	0.0165
H	4.1364	-0.4037	1.4075	H	-3.2858	1.8817	-1.3824
H	4.9138	-0.1137	-0.152	H	-2.0533	3.1404	-1.2505
H	4.1215	-2.031	-1.0131	H	-2.2139	3.1883	0.9894
H	3.287	-2.4505	0.4862	H	-3.435	1.9117	1.012
H	1.7965	-1.8297	-1.761	H	-0.9168	1.399	2.0432
H	3.2477	1.6846	-0.1741	H	-0.677	1.281	-2.0677
H	2.1661	1.5151	2.4966	H	-2.6967	-0.7163	-2.6217
H	0.7006	0.5527	2.6776	H	-2.9881	-1.8922	-1.3413
H	2.2771	-0.2423	2.6283	H	-3.7504	-0.3008	-1.2667
H	-0.214	-1.6465	1.6806	H	-3.0691	-1.8576	1.2573
H	0.3336	-2.8482	0.5129	H	-2.901	-0.6294	2.5103
H	1.454	-2.2231	1.7262	H	-3.8486	-0.2844	1.0611
H	-4.9274	0.4406	-0.6009	H	4.9235	-0.0121	-0.0133
H	-3.4636	-0.2817	-1.2369	H	4.3883	0.9755	-1.3624
H	-2.4032	0.2878	1.1284	H	3.285	-1.124	-1.5679
H	-3.8608	1.2174	1.399	H	2.0627	0.0405	-1.0982
H	-2.1573	2.7147	0.6357	H	2.3942	-0.793	1.3411
H	-3.3703	2.6007	-0.6313	H	3.4745	-2.0181	0.6843
H	-1.0789	2.6142	-1.5469	H	1.3113	-3.0322	0.9909
H	-1.9014	1.1102	-1.969	H	1.5606	-2.8696	-0.7602
H	-4.9492	-1.2386	1.2627	H	3.3422	1.2251	1.5157
H	-5.0043	-1.9808	-0.341	H	4.5434	2.2537	0.7479
H	-3.4527	-3.1606	0.8386	H	1.7293	1.9818	-0.0587
H	-2.5668	-2.2143	-0.1571	H	2.4182	3.245	0.6815
**Conf 3**	**X axis(Å)**	**Y axis(Å)**	**Z axis(Å)**	**Conf 4**	**X axis(Å)**	**Y axis(Å)**	**Z axis(Å)**
C	-2.7395	2.0814	-1.158	C	-2.7709	2.0478	-1.1851
C	-2.4528	2.5297	0.2787	C	-2.4738	2.527	0.2394
C	-1.3018	1.6071	0.6828	C	-1.3124	1.6208	0.651
C	-1.7469	0.1483	0.889	C	-1.7449	0.1638	0.8922
C	-2.0394	-0.304	-0.5579	C	-2.0507	-0.3202	-0.5419
C	-1.7114	0.9674	-1.3612	C	-1.7382	0.9358	-1.3749
C	-0.5355	-0.668	1.2911	C	-0.5233	-0.636	1.2969
N	-0.1581	-1.4637	0.2484	N	-0.1529	-1.4511	0.2666
C	-0.97	-1.3388	-0.8418	C	-0.9794	-1.3558	-0.8156
O	-0.8721	-1.9589	-1.892	O	-0.8929	-2.002	-1.8509
O	0.002	-0.6068	2.3886	O	0.026	-0.5504	2.3871
O	-0.6077	1.5163	-0.5909	O	-0.6309	1.5071	-0.627
C	-3.4042	-0.9165	-0.8482	C	-3.4156	-0.9452	-0.8041
C	-2.841	-0.0515	1.9306	C	-2.8257	-0.0219	1.9502
C	4.0388	-0.0241	-0.42	C	4.0495	-0.02	-0.4362
C	2.7722	-0.528	0.2754	C	2.77	-0.4983	0.2541

C	2.213	-1.7717	-0.4208	C	2.2161	-1.7603	-0.4128
C	0.987	-2.3547	0.2895	C	0.9963	-2.3359	0.3142
C	4.585	1.2253	0.2689	C	4.5838	1.2541	0.2162
N	5.7951	1.6852	-0.4034	N	5.8107	1.6851	-0.4444
H	-3.7745	1.7722	-1.3177	H	-3.8056	1.7296	-1.329
H	-2.5327	2.9148	-1.8399	H	-2.5752	2.8683	-1.8858
H	-2.1144	3.5726	0.2666	H	-2.1431	3.5718	0.2031
H	-3.325	2.4724	0.9332	H	-3.3394	2.4768	0.9033
H	-0.6475	2.0125	1.4591	H	-0.6532	2.0468	1.412
H	-1.4213	0.8032	-2.4023	H	-1.457	0.7513	-2.4151
H	-3.5214	-1.1157	-1.9196	H	-3.5438	-1.1667	-1.8699
H	-3.526	-1.874	-0.3286	H	-3.5267	-1.8924	-0.2638
H	-4.2297	-0.2682	-0.542	H	-4.2409	-0.2948	-0.5019
H	-3.0304	-1.117	2.105	H	-3.006	-1.0849	2.1481
H	-2.5467	0.3838	2.8925	H	-2.5232	0.4345	2.8997
H	-3.789	0.4082	1.6381	H	-3.78	0.4258	1.6596
H	4.7995	-0.815	-0.413	H	4.808	-0.8114	-0.3857
H	3.8138	0.1985	-1.4708	H	3.8456	0.1649	-1.4985
H	2.0221	0.2709	0.2738	H	2.0223	0.3021	0.2178
H	2.9966	-0.7623	1.3229	H	2.9788	-0.7018	1.3113
H	2.9907	-2.5444	-0.4551	H	2.9979	-2.5294	-0.4331
H	1.9608	-1.5258	-1.4595	H	1.959	-1.5382	-1.4557
H	1.232	-2.588	1.3315	H	1.249	-2.5508	1.3584
H	0.7047	-3.2893	-0.209	H	0.7154	-3.2803	-0.1664
H	3.8358	2.0247	0.2547	H	3.8393	2.0543	0.1446
H	4.8199	1.0063	1.316	H	4.7863	1.0805	1.2788
H	5.5778	1.9215	-1.3709	H	6.1747	2.5111	0.0286
H	6.1149	2.5479	0.0348	H	6.5234	0.9631	-0.3486
**Conf 5**	**X axis(Å)**	**Y axis(Å)**	**Z axis(Å)**	**Conf 6**	**X axis(Å)**	**Y axis(Å)**	**Z axis(Å)**
C	-2.4656	-2.2829	-0.4885	C	-3.906	-1.4566	-0.0602
C	-3.1127	-1.1965	-1.3531	C	-4.1762	-0.2339	0.8227
C	-2.044	-0.1028	-1.3514	C	-3.0829	0.7375	0.3748
C	-1.9085	0.5989	0.0126	C	-1.6783	0.3133	0.8382
C	-1.236	-0.4959	0.8682	C	-1.4062	-0.9179	-0.0531
C	-1.1159	-1.6563	-0.1348	C	-2.6991	-1.0024	-0.8828
C	-0.8499	1.676	-0.1037	C	-0.6796	1.3444	0.3541
N	0.282	1.2735	0.5449	N	0.0637	0.8151	-0.6612
C	0.1535	0.0476	1.1329	C	-0.2791	-0.4714	-0.9618
O	1.0116	-0.5465	1.7726	O	0.2396	-1.1792	-1.8147
O	-1.0074	2.7376	-0.6913	O	-0.5661	2.4685	0.8237
O	-0.8512	-0.9343	-1.3679	O	-3.0298	0.4039	-1.0387
C	-1.8836	-0.8569	2.199	C	-0.9937	-2.2139	0.6346
C	-3.1906	1.2275	0.5449	C	-1.5176	0.1479	2.3447

C	3.8242	-0.0093	-0.0705	C	4.436	0.2594	0.2315
C	2.7922	0.5491	-1.0551	C	3.0779	0.1855	-0.4703
C	2.3229	1.9648	-0.7019	C	2.4294	1.5685	-0.5761
C	1.4969	2.0687	0.588	C	1.1071	1.5518	-1.3501
C	4.1519	-1.4857	-0.31	C	5.0789	-1.1229	0.3334
N	3.0149	-2.3623	-0.0426	N	6.3608	-1.0343	1.0225
H	-3.069	-2.5647	0.3769	H	-3.7421	-2.3745	0.5083
H	-2.3167	-3.1826	-1.0973	H	-4.7693	-1.6214	-0.7158
H	-3.2529	-1.5851	-2.3688	H	-5.1651	0.1717	0.5779
H	-4.0886	-0.8726	-0.9852	H	-4.1664	-0.4596	1.8911
H	-2.0731	0.5603	-2.2199	H	-3.3271	1.793	0.5214
H	-0.3232	-2.3789	0.0761	H	-2.6022	-1.4942	-1.8543
H	-1.3602	-1.6961	2.6714	H	-0.9129	-3.031	-0.0916
H	-1.84	-0.0157	2.9005	H	-0.0128	-2.1121	1.1134
H	-2.9334	-1.143	2.0917	H	-1.7051	-2.5269	1.4036
H	-2.9988	1.8045	1.457	H	-0.4721	-0.0457	2.6114
H	-3.6182	1.9203	-0.189	H	-1.819	1.0611	2.8707
H	-3.9567	0.4839	0.7809	H	-2.1173	-0.6746	2.7438
H	4.749	0.574	-0.1618	H	5.0975	0.9377	-0.3222
H	3.4874	0.1131	0.9639	H	4.3052	0.6836	1.2352
H	1.923	-0.11	-1.1347	H	2.4233	-0.4924	0.0893
H	3.2458	0.5738	-2.0541	H	3.206	-0.2383	-1.4736
H	1.7225	2.3416	-1.5394	H	3.1192	2.2494	-1.0895
H	3.1936	2.6263	-0.6194	H	2.2688	1.9746	0.43
H	1.2075	3.1168	0.729	H	1.26	1.1219	-2.3462
H	2.0939	1.7739	1.4564	H	0.7626	2.5847	-1.4771
H	4.4978	-1.6401	-1.3377	H	4.4233	-1.8039	0.8866
H	4.9731	-1.7782	0.3533	H	5.2316	-1.5446	-0.6661
H	2.5692	-2.0701	0.8336	H	6.8009	-1.9536	1.0353
H	2.3012	-2.2218	-0.7568	H	6.9899	-0.4256	0.5002
**Conf 7**	**X axis(Å)**	**Y axis(Å)**	**Z axis(Å)**	**Conf 8**	**X axis(Å)**	**Y axis(Å)**	**Z axis(Å)**
C	2.9214	-1.7144	-1.372	C	2.3868	2.2398	0.5751
C	2.4964	-2.4795	-0.1145	C	2.4087	2.0967	-0.9495
C	1.2638	-1.6966	0.3406	C	1.2917	1.0821	-1.1968
C	1.6115	-0.312	0.9155	C	1.6625	-0.3424	-0.7446
C	2.0444	0.4596	-0.3498	C	1.6529	-0.201	0.7931
C	1.8706	-0.6057	-1.4472	C	1.2662	1.2795	0.9744
C	0.3242	0.3666	1.3371	C	0.4815	-1.2446	-1.0381
N	0.0318	1.3701	0.4593	N	-0.1538	-1.5511	0.1302
C	0.9663	1.5091	-0.526	C	0.4766	-1.0472	1.2314
O	0.9581	2.3459	-1.4186	O	0.1529	-1.2299	2.3969
O	-0.3329	0.0458	2.3183	O	0.1591	-1.6157	-2.1584
O	0.7146	-1.3373	-0.9561	O	0.3636	1.4796	-0.1495

C	3.4026	1.1502	-0.3303	C	2.8947	-0.6433	1.5561
C	2.5691	-0.3272	2.1006	C	2.908	-0.9255	-1.3985
C	-4.0028	0.02	-0.9745	C	-4.0408	0.4969	-0.5366
C	-2.8374	0.3863	-0.0505	C	-2.8858	-0.4801	-0.7779
C	-2.2644	1.7632	-0.3993	C	-2.5992	-1.3858	0.4251
C	-1.1513	2.2054	0.5564	C	-1.3916	-2.3059	0.2021
C	-4.5402	-1.3936	-0.7417	C	-3.7758	1.5787	0.5163
N	-5.151	-1.5434	0.5742	N	-2.6743	2.4685	0.1715
H	3.9518	-1.355	-1.3338	H	3.3493	2.0268	1.0449
H	2.8329	-2.3779	-2.2405	H	2.1114	3.2693	0.8333
H	2.2125	-3.5004	-0.3965	H	2.1503	3.0611	-1.4027
H	3.2854	-2.5511	0.6371	H	3.3821	1.7961	-1.3428
H	0.5463	-2.2802	0.9235	H	0.8246	1.1547	-2.1826
H	1.6923	-0.2181	-2.4538	H	0.7751	1.5243	1.92
H	3.6311	1.5871	-1.3092	H	2.7987	-0.4128	2.6233
H	3.4189	1.9691	0.3983	H	3.0439	-1.7261	1.4736
H	4.2179	0.4673	-0.0766	H	3.8048	-0.1566	1.1948
H	2.6859	0.676	2.5268	H	3.0481	-1.9749	-1.1148
H	2.1885	-0.9732	2.9001	H	2.8241	-0.8967	-2.491
H	3.5652	-0.689	1.8318	H	3.8183	-0.3852	-1.1244
H	-4.8137	0.7493	-0.8538	H	-4.2864	0.9833	-1.4895
H	-3.6693	0.0913	-2.0173	H	-4.9337	-0.0684	-0.2422
H	-2.0568	-0.3772	-0.1417	H	-1.9925	0.0811	-1.0667
H	-3.1709	0.3911	0.9933	H	-3.1422	-1.1129	-1.637
H	-3.069	2.5076	-0.3591	H	-3.4819	-2.0065	0.6207
H	-1.8893	1.7524	-1.4299	H	-2.4292	-0.7814	1.3223
H	-1.5182	2.2007	1.5888	H	-1.5236	-2.8913	-0.7142
H	-0.8611	3.2321	0.3052	H	-1.3162	-3.0007	1.0463
H	-5.2964	-1.6148	-1.5027	H	-4.681	2.1872	0.6229
H	-3.7356	-2.1281	-0.8586	H	-3.5878	1.1332	1.4978
H	-5.4961	-2.4971	0.6761	H	-1.783	1.9628	0.1759
H	-4.4414	-1.427	1.2972	H	-2.7855	2.8052	-0.7836

Table S5. The Cartesian coordinates of the lowest energy conformers for (1*R*,2*R*,3*S*,6*R*)-**5**

Conf 1	X axis(Å)	Y axis(Å)	Z axis(Å)	Conf 2	X axis(Å)	Y axis(Å)	Z axis(Å)
C	-2.3737	-1.2361	-0.0953	C	-1.3461	2.1305	-0.3788
C	-2.7005	0.186	0.372	C	0.0328	2.6784	0.003
C	-1.5302	0.9879	-0.1994	C	0.9596	1.5253	-0.3844
C	-0.2019	0.6879	0.5179	C	0.8229	0.309	0.544
C	0.1288	-0.7143	0.0031	C	-0.558	-0.2293	0.1662
C	-1.0806	-1.0302	-0.8874	C	-1.0031	0.7461	-0.9313
C	0.8776	1.5399	-0.0868	C	1.7604	-0.7616	0.0584
O	1.7031	0.8077	-0.8658	O	1.0794	-1.7969	-0.4779

C	1.3088	-0.4838	-0.8986	C	-0.2476	-1.556	-0.461
O	1.8485	-1.3467	-1.5776	O	-1.0924	-2.3439	-0.8642
O	0.9986	2.7433	0.0924	O	2.9773	-0.7246	0.1596
O	-1.3493	0.2801	-1.4537	O	0.2693	1.044	-1.5678
C	0.507	-1.8039	0.9948	C	-1.5627	-0.4747	1.2848
C	-0.2424	0.925	2.0237	C	1.1048	0.5767	2.017
O	1.5953	-1.3745	1.8034	O	-2.8644	-0.709	0.7587
H	-2.2884	-1.9516	0.725	H	-2.0508	2.1157	0.4558
H	-3.1725	-1.5861	-0.7598	H	-1.7754	2.7581	-1.1686
H	-3.6408	0.5075	-0.0915	H	0.2495	3.5602	-0.6116
H	-2.8181	0.2706	1.4546	H	0.1049	2.9783	1.0506
H	-1.7458	2.046	-0.3702	H	1.9839	1.8261	-0.6199
H	-0.9091	-1.771	-1.673	H	-1.7214	0.3525	-1.6553
H	-0.3178	-2.0497	1.6686	H	-1.2868	-1.3337	1.9073
H	0.8053	-2.7235	0.4786	H	-1.6482	0.3913	1.947
H	-0.691	1.9001	2.2479	H	2.1035	1.0091	2.1492
H	-0.8269	0.1641	2.5492	H	0.3873	1.2704	2.4636
H	0.762	0.9385	2.4609	H	1.0773	-0.3508	2.6005
H	1.9143	-2.1486	2.3039	H	-2.8411	-1.5484	0.2532

Conf 3	X axis(Å)	Y axis(Å)	Z axis(Å)
C	2.358	-0.2397	-0.9354
C	1.9887	-1.6061	-0.3516
C	0.4633	-1.6096	-0.4711
C	-0.2225	-0.6775	0.5382
C	0.1547	0.7086	0.0062
C	0.9884	0.3624	-1.2396
C	-1.7068	-0.7452	0.297
O	-2.1681	0.4046	-0.2396
C	-1.1569	1.2694	-0.46
O	-1.306	2.3813	-0.9461
O	-2.4206	-1.6988	0.5709
O	0.3085	-0.8413	-1.6963
C	0.756	1.7429	0.9518
C	0.0288	-1.0036	2.0052
O	1.8847	1.2472	1.6512
H	2.9896	0.3639	-0.2828
H	2.912	-0.3941	-1.8696
H	2.4203	-2.3931	-0.9812
H	2.3578	-1.7565	0.6649
H	0.019	-2.6034	-0.5716
H	0.997	1.1233	-2.0247
H	1.0688	2.6347	0.3959
H	0.0197	2.0643	1.6981

H	-0.3258	-2.0131	2.2451
H	1.0858	-0.9694	2.2775
H	-0.5106	-0.3119	2.6624
H	2.2307	1.9746	2.2011
